# Independent allopatric polyploidizations shaped the geographical structure and initial stage of reproductive isolation in an allotetraploid fern, *Lepisorus nigripes* (Polypodiaceae)

**DOI:** 10.1371/journal.pone.0233095

**Published:** 2020-05-20

**Authors:** Tao Fujiwara, Yasuyuki Watano

**Affiliations:** 1 Center for Integrative Conservation, Xishuangbanna Tropical Botanical Garden, Chinese Academy of Sciences, Mengla, Yunnan, China; 2 Department of Biology, Graduate School of Science, Chiba University, Chiba, Japan; National Cheng Kung University, TAIWAN

## Abstract

Although polyploidy is pervasive and its evolutionary significance has been recognized, it remains unclear how newly formed polyploid species become established. In particular, the impact of multiple origins on genetic differentiation among populations of a polyploid species and whether lineages of independent origins have different evolutionary potentials remain open questions. We used population genetic and phylogenetic approaches to identify genetic differentiation between lineages with independent origins within an allotetraploid fern, *Lepisorus nigripes*. A total of 352 individuals from 51 populations were collected throughout the distribution range. To examine the genetic structure, multilocus genotyping, Bayesian population structure analysis, and neighbor-net analysis were carried out using single-copy nuclear genes. Phylogenetic trees were constructed to detect recurrent polyploid origins. Proportions of abortive spores were analysed as the measure of postzygotic reproductive isolation. Two genetically distinct lineages, the East-type and the West-type, were distributed mainly in the eastern and western parts, respectively, of the Japanese archipelago. Phylogenetic analyses indicated independent origins of these types and detected additional independent origins within each type. We also revealed limited genetic recombination between both types, even in their sympatric regions. F_1_ hybrids between the East- and West-types showed a reduction in fertility. It is likely that the East- and West-types formed independently in the eastern and western parts of Japan, respectively. The limited genetic recombination and reduced fertility of hybrids suggest that the two types are at an incipient stage of speciation. Two polyploid lineages with independent geographic origins could develop reproductive isolation barrier(s).

## Introduction

Polyploidy or whole-genome duplication is prevalent in all eukaryotic species, including plants, animals, and fungi, and this process plays an important role in their diversification and evolution [1,2]. Polyploidy is a particularly significant process in the plant kingdom. During the last two decades, researchers have revealed that most lineages of vascular plants, including ferns, have experienced at least one whole-genome duplication event, namely an ancient polyploidization event [3–5], respectively. In addition, many contemporary species are of recent polyploid origin [6]. Although the evolutionary significance of polyploidy has been widely recognized, some aspects of its evolutionary consequences remain poorly resolved [7–10]. In particular, because recently formed polyploid lineages show the reduction in net diversification rate comparing with those that the closely related diploid lineages show, most of them are thought to be evolutionary dead ends [11,12]. Thus, further understanding of how nascent polyploid individuals increase their number and become established as species is needed to evaluate the evolutionary consequences of polyploidy. Because chromosome doubling directly serves as a postzygotic barrier against parental species, polyploid speciation is believed to be an example of non-ecological and sympatric speciation [13]. However, recent studies suggest that polyploid speciation might also require the development of prezygotic barriers to avoid reproductive interference by parental species, which include a transition to selfing and niche differentiation [10,12,14–16].

Another problem that we focus on in this paper is the evolutionary consequences of multiple origins. In the traditional view, each polyploid species was considered to have originated only once (a single origin). This is because polyploidization was regarded as a very rare event [17]. Therefore, Stebbins, who developed central tenets of polyploid evolutionary thinking in the 20^th^ century, viewed polyploid species as genetically depauperate with limited evolutionary potential [17,18]. Through the advent of molecular approaches, however, it has revealed that many polyploid species originated recurrently from different parental genotypes [19,20]. Multiple origins contribute to an increase in genetic diversity within polyploid species; the introduced genetic diversity is considered to facilitate ecological adaptation under a new genetic/genomic background and to lead to the evolutionary success of polyploid species [21]. For two decades, however, whether independent lineages with different origins interbreed with each other or have different evolutionary potentials, ultimately forming cryptic species has remained an open question [2,9,22]. Symonds et al. [23] hypothesized that the outcomes of multiple origins in polyploid species range from a single homogeneous species to multiple genetically distinct lineages that ultimately form cryptic species, owing to genetic variations contributed by parent species and the degree of gene flow among lineages with independent origins. In fact, a few studies have indicated no or limited gene flow among populations of independent origins [23,24]. For example, Perrie et al. [24] conducted a population genetic analysis of allooctoploid *Asplenium* ferns in New Zealand using the AFLP method. They found that allopolyploid lineages with independent origins remained genetically distinct even when sympatric and they called this phenomenon “parallel polyploid speciation”. Currently, however, only a few researchers have examined genetic differentiation and gene flow between independently formed polyploid lineages within a species [23–25].

Homosporous ferns provide ideal research materials for understanding polyploid speciation because they show a high frequency of polyploid speciation among vascular plants [6]. This may be partly due to the inherent characteristics of the homosporous plant life cycle. In homosporous ferns, a polyploid sporophyte can be formed in a single step through selfing within a hermaphrodite gametophyte from an unreduced spore that is produced by sterile hybrids [26]. Additionally, allozyme analyses revealed that congeneric fern species are characterized by lower interspecific genetic identities (namely, higher interspecific genetic distances) than seed plants [27]. The genetic distinctness between fern species seems to work in favour of reconstructing reticulate evolutionary processes involving polyploidization.

In a previous study [28], an allopolyploid epiphytic fern, *Lepisorus nigripes* T.Fujiw. & Seriz., was discriminated from *L*. *thunbergianus* sensu lato and described as a new species. This species is endemic to Japan and is widely distributed throughout the Japanese archipelago, from Hokkaido Island to Kyushu Island. Allozyme analysis [29] and nuclear gene phylogeny [28] indicated that *L*. *nigripes* is an allotetraploid species (2*n* = 100 and 102) between *L*. *thunbergianus* (Kaulf.) Ching (2*n* = 50) and *L*. *angustus* Ching (2*n* = 52). *Lepisorus nigripes* and its parental species show different habitat preferences. The difference in geographical distribution pattern between diploid (*L*. *thunbergianus* sensu strict) and tetraploid (*L*. *nigripes*) cytotypes has been well-studied throughout the mainlands of Japan [30–32]. *Lepisorus thunbergianus* sensu stricto is distributed in coastal and lowland regions of the southern part of Japan, from Kanto district to Okinawa Pref., while *L*. *nigripes* has a more northerly geographic distribution from Hokkaido Island to Kyushu Island and tends to grow more inland and in higher altitude areas at the same latitude [32]. However, the two species frequently co-occur and produce sterile triploid hybrids [31]. *Lepisorus angustus*, the other parental species of *L*. *nigripes*, has a relatively more restricted distribution, growing at elevations ranging from 1000 to 2000 m in the mountainous region of Honshu Island [33]. This species never co-occurs with *L*. *thunbergianus* and *L*. *nigripes*. Phylogenetic analysis had indicated that *L*. *nigripes* originated recurrently, at least four times [28]. Extant diploid progenitors and its wide distribution in the Japanese archipelago make *L*. *nigripes* one of the ideal fern models to examine the evolutionary consequences of multiple origins.

Here, we employ population genetic approaches using single-copy nuclear genes to show the population genetic structure of *L*. *nigripes* throughout its distributional range. The results showed a clear pattern of genetic differentiation between the eastern and western parts of the Japanese archipelago, which is a phylogeographic pattern that is frequently observed in both evergreen and deciduous broad-leaved trees [34,35], but found for the first time in ferns. We address 1) whether the East-and West-types of *L*. *nigripes* have separate polyploid origins; 2) the extent of hybridization and recombination between the two types in sympatric regions and 3) whether F_1_ hybrids between the two types show a reduction in fertility; and we present 4) a possible scenario to explain the East-West pattern of differentiation in this allotetraploid fern species.

## Materials and methods

### Plant collection

To cover its known distribution range, a total of 352 samples of *Lepisorus nigripes* were collected from 51 populations (Table 1 and S1 Table). No permits for collections were required because our all collection sites did not belong to nature reserve such as Japanese National forest and the plant material did not involve endangered or protected species. *Lepisorus nigripes* is morphologically similar to its parental diploid species, *L*. *thunbergianus* [28]. Additionally, these two species frequently co-occur and readily hybridize with each other, producing triploid hybrids [31]. To avoid the inclusion of triploid hybrids in our samples, spore morphology was used. Sporangia of the triploid hybrids are highly abortive and hardly produce viable spores. Therefore, we removed individuals with abortive sporangia. For phylogenetic analyses, the two parental species, *L*. *angustus* and *L*. *thunbergianus*, and closely related species, *L*. *hachijoensis* Sa. Kurata, *L*. *kuratae* T. Fujiw. & Seriz., *L*. *onoei* (Franch. et Sav.) Ching, *L*. *tosaensis* (Makino) H. Itô, and *L*. *uchiyamae* (Makino) H. Itô, were collected at several localities (S2 Table). *Lepisorus kuratae* is an allotetraploid of hybrid origin between *L*. *thunbergianus* and an unknown diploid race of *L*. *tosaensis* and was described as a new species jointly with *L*. *nigripes* [28].

**Table 1:** Numbers of East- and West-type individuals, inbreeding coefficients (*F*), and allelic richness (*A*r) in the *Lepisorus nigripes* populations.

Region	Pop. code	No. of samples					No. of MLGs^$^				*F*			*A*_R_^#^		
		total	E	W	F_1_	R	E	W	F_1_	R	E	W	MIX	E	W	MIX
Northern Honshu															
	1	4	4	0	0	0	3	0	0	0	-	-	-	-	-	-
	2	11	11	0	0	0	3	0	0	0	**0.848**	-	-	1.26	-	-
	3	10	9	0	0	1	1	0	0	1	NA	-	1.000	1.00	-	1.14
	4	8	8	0	0	0	3	0	0	0	0.236	-	-	1.49	-	-
	5	7	3	0	0	4	2	0	0	1	-	-	**1.000**	-	-	1.50
	6	6	6	0	0	0	3	0	0	0	**1.000**	-	-	1.33	-	-
	all	46	41	0	0	5	8	0	0	2				1.77	-	2.01
Central Honshu																
	7	3	3	0	0	0	1	0	0	0	-	-	-	-	-	-
	8	5	5	0	0	0	1	0	0	0	-	-	-	-	-	-
	9	6	6	0	0	0	2	0	0	0	**1.000**	-	-	1.33	-	-
	10	11	11	0	0	0	6	0	0	0	**0.757**	-	-	1.49	-	-
	11	10	10	0	0	0	5	0	0	0	**1.000**	-	-	1.48	-	-
	12	4	4	0	0	0	2	0	0	0	-	-	-	-	-	-
	13	8	8	0	0	0	1	0	0	0	NA	-	-	1.00	-	-
	14	7	7	0	0	0	1	0	0	0	NA	-	-	1.00	-	-
	15	7	7	0	0	0	2	0	0	0	0.000	-	-	1.29	-	-
	16	3	1	2	0	0	1	1	0	0	-	-	-	-	-	-
	17	7	6	0	1	0	1	0	1	0	NA	-	0.000	1.00	-	1.71
	18	7	1	5	1	0	1	3	1	0	-	-	**0.722**	-	-	2.31
	all	78	69	7	2	0	11	4	2	0				1.70	1.33	2.25
Western Honshu																
	19	6	0	6	0	0	0	1	0	0	NA	-	-	-	1.00	-
	20	3	3	0	0	0	1	0	0	0	-	-	-	-	-	-
	21	8	3	0	0	5	1	0	0	1	-	-	**1.000**	-	-	1.50
	22	28	21	4	1	2	2	2	1	2	1.000	-	**0.820**	1.08	-	1.75
	23	7	0	7	0	0	0	3	0	0	-	**1.000**	-	-	1.33	-
	24	8	0	8	0	0	0	2	0	0	-	**1.000**	-	-	1.17	-
	25	8	4	3	0	1	2	1	0	1	-	-	**0.949**	-	-	1.98
	26	7	3	1	2	1	2	1	2	1	-	-	0.143	-	-	1.83
	27	4	4	0	0	0	2	0	0	0	-	-	-	-	-	-
	28	5	5	0	0	0	1	0	0	0	-	-	-	-	-	-
	29	6	4	2	0	0	1	2	0	0	-	-	**1.000**	-	-	1.83
	30	7	1	3	2	1	1	2	1	1	-	-	0.318	-	-	1.83
	31	3	2	1	0	0	1	1	0	0	-	-	-	-	-	-
	32	4	2	1	0	1	1	0	0	1	-	-	-	-	-	-
	33	4	1	2	0	1	1	1	0	1	-	-	-	-	-	-
	34	7	0	7	0	0	0	2	0	0	-	1.000	-	-	1.17	-
	35	4	0	4	0	0	0	3	0	0	-	-	-	-	-	-
	36	4	0	4	0	0	0	1	0	0	-	-	-	-	-	-
	37	5	0	5	0	0	0	1	1	0	-	-	-	-	-	-
	38	3	0	3	0	0	0	2	0	0	-	-	-	-	-	-
	39	9	0	6	3	0	0	1	2	0	-	NA	-0.055	-	1.00	1.76
	all	140	53	67	8	12	4	9	4	6				1.21	1.67	2.29
Region	Pop. code	No. of samples					No. of MLGs^$^				*F*^#^			*A*_R_^$^		
		total	E	W	F_1_	R	E	W	F_1_	R	E	W	MIX	E	W	MIX
Shikoku																
	40	8	0	6	2	0	0	1	1	0	-	NA	-0.077	-	1.00	1.63
	41	8	0	7	1	0	0	2	1	0	-	1.000	0.317	-	1.16	1.66
	42	10	5	5	0	0	2	2	0	0	-	-	**1.000**	-	-	1.95
	43	5	3	1	0	1	1	1	0	1	-	-	-	-	-	-
	44	5	0	2	1	2	0	1	1	2	-	-	-	-	-	-
	45	5	0	5	0	0	0	3	0	0	-	-	-	-	-	-
	46	10	0	10	0	0	0	3	0	0	-	0.250	-	-	1.17	-
	all	51	8	36	4	3	2	6	1	3				1.16	1.28	2.00
Kyushu																
	47	6	0	6	0	0	0	3	0	0	-	0.706	-	-	1.17	-
	48	8	0	8	0	0	0	3	0	0	-	0.632	-	-	1.17	-
	49	11	0	11	0	0	0	2	0	0	-	1.000	-	-	1.16	-
	50	5	0	5	0	0	0	2	0	0	-	-	-	-	-	-
	51	7	0	7	0	0	0	1	0	0	-	NA	-	-	1.00	-
	all	37	0	37	0	0	0	5	0	0				-	1.29	-
Mean											0.730	0.824	0.581	1.23	1.13	1.74

MLG, multilocus genotype; E, East-type; W, West-type; F_1_, F_1_ individuals between the East- and West-types; R, Recombinant individual (later generation hybrid or backcrossed individual) between the East- and West-types; MIX, population with both East- and West-type individuals.

$, MLGs with missing data were not counted.

#, Allelic richness values were calculated based on the minimum sample size of six for individual populations, and seven for regions.

### DNA extraction, PCR amplification and nucleotide sequencing

Total genomic DNA was extracted from a silica gel-dried sample using the CTAB method [36]. A chloroplast DNA region, an intergenic spacer between *rps4* and *trnS* (hereafter r*ps4-trnS*), was amplified using the primers rps4_PTER_F and trnS_PTER_R1 [28]. For single-copy nuclear genes, we selected three genes, *PgiC* (cytosolic phosphoglucose isomerase), *GapCp* (NAD-dependent glyceraldehyde-3-phosphate dehydrogenase) and *pTPI* (plastidic triose phosphate isomerase). Intron 15 of *PgiC* was amplified using the primers reported by Fujiwara et al. [28]. For *GapCp*, we used a forward primer, GAPCP_ POL_8F, that was newly designed based on the sequence of *Polypodium amorphum* Suksd. (KJ748228) presented in Sigel et al. [37], and a reverse primer (ESGAPCP11R1) reported in Schuettpel*z* et al. [38] to amplify a region from intron 8 to intron 10 of the gene. For *pTPI*, we developed a new primer set to amplify intron 5 of the gene based on the sequence of scaffold-YLJA-2013048-Polypodium_amorphum, which was obtained by a BLASTn search of the 1KP project database using the AT2G21170 gene of *Arabidopsis thaliana* (L.) Heynh. as a query. Primers used for PCR amplification and sequencing are listed in Table 2. PCR thermocycling conditions involved initial denaturation at 94°C for 3 min followed by 35 cycles at 94°C for 45 s, 56°C for 45 s (*rps4-trnS*) or 54°C for 45 s (nuclear genes), 72°C for 90 s, and a final extension at 72°C for 7 min.

**Table 2:** Primers used for PCR amplification and sequencing of the chloroplast DNA and nuclear genes.

Gene or region	Primer	5’-3’ Primer sequence	Primer source
*rps4-trnS*	Rps4_Pter_F	CTCGCTACCGAGGACCTCG	Fujiwara et al. [28]
	TrnS_Pter_R	CTACCGAGGGTTCGAATC	Fujiwara et al. [28]
*PgiC*	PGIC_LEP_15F	TTGCCAGGCATTAGAGAAGC	Fujiwara et al. [28]
	PGIC_LEP_16R	GCCTTCTATTGAAACCCCCTTTC	Fujiwara et al. [28]
*GapCp*	GAPCP_POL_8F	ATCATCCCAAGCTCAACTGG	This study
	ESGAPCP11R1	GTATCCCCAYTCRTTGTCRTACC	Schuettpelz et al. [38]
*pTPI*	PTPI_POL_5F	ATTGCATGTGTGGGTGAGAA	This study
	PTPI_POL_6R	GCTTGAGGCGAGACATTCTT	This study

In order to infer genotype at the nuclear loci for all individuals and to determine the sequences of alleles separated on gels, we conducted single-strand conformation polymorphism (SSCP) gel electrophoresis of PCR products, generally following the methods of Jaruwattanaphan et al. [39] and Fujiwara et al. [40]. For determining DNA sequence of each of alleles, a part of the DNA band separated on the SSCP gel was excised, and DNA extracted from the band was used as a template for further PCR amplification [39]. Per each of DNA bands considered to represent different allele, multiple bands from individuals collected in different populations were sequenced.

For DNA sequencing, the PCR products were purified using Illustra ExoStar 1-Step (GE Healthcare, Chicago, Illinois, USA) and used as templates for direct sequencing. Cycle sequencing was conducted with a BigDye Terminator version 3.1 cycle sequencing kit (Applied Biosystems, Foster City, California, USA). The sequencing products were analysed by an ABI3500 genetic analyser (Applied Biosystems) and also partly by Eurofins Genomics (Tokyo, Japan). The resulting nucleotide sequences were deposited into the International Nucleotide Sequence Databases (INSD) (see S3 Table).

### Multilocus genotype analyses

For assignment of homoeologous loci (a pair of loci derived from two parental genomes) into each sub-genome, phylogenetic approach is commonly performed. However, this approach is inapplicable in the case that a gene phylogeny exhibits incomplete lineage sorting between parental species. In our study, as shown in the results of phylogenetic analysis, incomplete lineage sorting between *Lepisorus thunbergianus* and *Lepisorus angustus* was highly expected. To avoid this problem, we calculated genetic distances between DNA sequences from allotetraploids and those of *L*. *angustus*, based on Kimura’s two parameter model [41] implemented in MEGA7 [42]. For individuals with only two sequences (putative homozygote at both homoeologous loci), the one closer to *L*. *angustus* sequences was considered to be coded at the *L*. *angustus*-derived locus (designated as the “*nigripes-A*” locus), and the other at the *L*. *thunbergianus*-derived locus (designated as the “*nigripes-T*” locus). Although some *L*. *nigripes* individuals had three or four different sequences, all of these sequences were found in the individuals having only two sequences. Therefore, each of these sequences could be assigned to the “*nigripes-A*” locus or the “*nigripes-T*” locus based on the results for the individual having only two sequences. With respect to *L*. *nigripes*, alleles at the “*Ang*” locus were labelled as *nigripes-A1*, *nigripes-A2*, and so on, and those at the “*nigripes-T*” locus as *nigripes-T1*, *nigripes-T2*, and so on. Alleles from progenitor diploid species were named allele 1, allele 2, and so on. Alleles from the other *Lepisorus* species were coded alphabetically if multiple sequences were obtained. The assignment was confirmed phylogenetically for each gene as described in the Results section.

Allelic richness (*A*r) values were calculated to show the genetic diversity for each population using FSTAT v. 2.9.3 [43]. The level of inbreeding was estimated for each population by calculating Wright’s fixation index (*F*), as implemented in FSTAT. Analysis of molecular variance (AMOVA) [44] was carried out to analyse the hierarchical population genetic structure using GenAlEx 6.5 [45]. *A*r and *F* value estimations and AMOVA were conducted only for the populations that had more than five individuals.

Population genetic structure analysis was also conducted using the Bayesian clustering method based on multilocus genotypes (MLGs) at six nuclear loci (three pairs of homoeologous loci). Masuyama et al. [46] suggested a high gametophytic selfing ability of *L*. *nigripes* (“the tetraploids of *L*. *thunbergianus*” in the original paper) based on the observation that nearly all of the gametophytes can form sporophytes in isolated culture condition. In fact, as mentioned in the Results section of this paper, genotype data showed an excess of homozygosity, suggesting that *L*. *nigripes* is a predominantly selfing species. Although STRUCTURE software [47] is the most commonly used software for Bayesian genetic clustering, it assumes random mating within population; therefore, we employed InStruct [48], which does not assume random mating and can also infer selfing rates for each cluster. The model was run with the likely number of clusters (*K*) set to values from 1 to 10, using a burn-in of 300,000 MCMC iterations followed by 600,000 MCMC iterations, and 15 independent chains were run for each *K*. Subsequently, we chose 10 runs in 15 runs based on the likelihood value to remove runs suspected to be outliers. Because total number of individuals per population was variable (from N = 3 to 28) and such a uneven sampling leads to wrong inference of population structure and misestimating of cluster number [49], we performed the correction method proposed by Puechmaille [49] in addition to the commonly used method, Δ*K* method [50] to determine optimal K in *L*. *nigripes* populations for two different datasets. For this, we conducted Instruct analysis for 1) a full dataset (total number of populations: 51 and total number of individuals: 352) and 2) trimmed dataset for which six individuals were randomly selected from each population with N ≥ 6 after removing the population with N < 6 (total number of populations: 31 and total number of individuals: 186). Subsequently, using the outputs from Instruct result for each dataset, we calculated Δ*K* and the corrected estimators for optimal K determination that is robust to uneven sampling, MedMeaK, MaxMeaK, MedMedK, and MaxMedK with threshold value 0.8 [49]. Cluster assignments were executed using the CLUMPP program [51], and the result was illustrated using the Distruct program [52]. We plotted the result for each population at K = 2 in map made by GeoMapApp (http://www.geomapapp.org/) [53] / CC BY / CC BY (Ryan et al. [54]). In order to discriminate between pure and admixed individuals at the optimal number of *K*, we used the results of cluster assignments (Q-matrix) generated by InStruct. We assigned individuals with q values of 95% and higher to non-admixed ones and those with q < 95% to admixed ones. Additionally, NewHybrids [55] was used to classify individuals into pure individuals, F_1_, F_2_ or backcrosses based on the default genotype categories. The analysis was run for 50,000 sweeps after a burn-in period of 10,000 sweeps.

Agglomerate clustering of MLGs was performed using the neighbor-net method [56], which was implemented using the SplitsTree4 application [57]. Pairwise genetic distance matrices among MLGs were generated using Kosman & Leonard’s similarity index [58], as implemented in the R package PopGenReport [59].

### Phylogenetic analyses

To detect lineages with independent origin, we conducted phylogenetic analyses for cpDNA and nuclear genes. The number of lineages with independent origins was interpreted as the number of the lineages with different alleles independently derived from different individuals of parental species. Because the transmission of alleles from parental species into polyploid is independent among different gene, the use of species tree combining multiple genes can lead to under- or overestimation of the number of origins. Thus, we separately reconstructed each gene phylogeny. The sequences determined in the present study and those generated in a previous study [28] were aligned using MUSCLE [60] implemented in AliView [61], and the resultant multiple alignment was edited manually in AliView. INSD accession numbers of the sequences used are shown in S3 Table. For chloroplast DNA and nuclear genes, indels were coded as binary data using the “simple index coding” method [62] using the IndelCoder option of SeqState 1.4.1 software [63]. Binarized characters were added to the data matrix, and the corresponding nucleotide sites with gaps were excluded as missing data. Only one sequence for each haplotype was included in each dataset. For phylogenetic analysis, each of the plastid regions, *rps4-trnS*, and nuclear genes, *PgiC*, *GapCp*, and *pTPI*, were separately analysed by the maximum likelihood (ML) method using Garli 2.01 [64] and Bayesian inference (BI) using MrBayes v 3.1.2 [65]. The best fitting substitution model for each DNA region was determined using the Akaike information criterion (AIC) [66] in jModelTest [67]. For binary-coded data, the Mkv model [68] was applied. In the ML analysis, eight independent runs were performed with randomly started trees and ‘genthreshfortopoterm’ set to 100,000. The maximum likelihood bootstrap support (MLBS) values were assessed using 500 bootstrap pseudo-replicate data sets. The consensus tree of bootstrap replicates was obtained using SumTrees [69]. In the BI analysis, four MCMC chains were run for 3,000,000 generations with samples taken every 100 generations. For evaluating convergence, Tracer 1.6 [70] was used. The first 500,000 generations were discarded as burn-in.

### Observation of spore fertilities

Spore fertility was evaluated by the proportion of normal spores per sporangium. Samples were selected based on the result of genetic clustering by InStruct: 11 F_1_ individuals (from six populations) between different genetic clusters, ten non-admixed individuals (from six populations) with heterozygous genotypes, and 17 non-admixed individuals (from 17 populations) with a homozygous genotype. At least five sporangia per sample were examined. Each sporangium was transferred to a glycerol solution (glycerol: water = 3: 1) on a glass slide, and spores were forcibly discharged from each sporangium using a dissecting needle. Shrunken small spores and transparent ones were interpreted as abnormal spores based on the criteria presented by Masuyama *et al*. [71]. The differences in the spore fertilities among F_1_ hybrids and heterozygous, and homozygous non-admixed individuals were tested using the Steel-Dwass test performed in R [72].

## Results

### Multi-locus genotyping and population genetic structure

In nuclear DNA markers, nearly all of the samples showed heterozygous SSCP banding patterns at all three nuclear genes, *PgiC*, *GapCp*, and *pTPI*. Mean pairwise genetic distances between each sequence from the samples of *L*. *nigripes* and those from *L*. *angustus* were calculated for each gene (Table 3). Each of the sequences found in *L*. *nigripes* was successfully assigned to the “*nigripes-A*” locus or the “*nigripes-T*” locus; there was a gap between the distances for the sequences of the “*nigripes-A*” locus and those of the “*nigripes-T*” locus (Table 3). Finally, we obtained sequences at six loci: two homoeologous loci for each of *PgiC*, *GapCp*, and *pTPI*. Polymorphisms were found in all loci examined. The number of alleles at each locus ranged from two (*PgiC*-*nigripes-A*, *pTPI-nigripes-T*, *pTPI*-*nigripes-A*) to 12 (*PgiC*- *nigripes-T*) (Table 3). Combining genotypes at six nuclear loci yielded 51 multi-locus genotypes (MLGs) in a total of 352 samples (S4 and S5 Tables). As for cpDNA, we found five haplotypes (A, B, C, D1, and D2) among the sequences determined from the 62 representative individuals: one or two from each of 51 locations (S4 Table).

**Table 3:** The length of sequences obtained from *Lepisorus nigripes*, pairwise genetic distances compared to *L*. *angustus* sequences, and observed number of alleles (*A*o) at each locus.

Locus	Length (bp)	Pairwise genetic distance against *L*. *angustus* sequences	*A*o
*PgiC-nigripes-T*	455–513	0.070–0.088	12
*PgiC- nigripes-A*	541	0.044	2
*GapCp-nigripes-T*	592–593	0.028–0.041	4
*GapCp-nigripes-A*	590–591	0.009–0.023	3
*PTPI-nigripes-T*	261–262	0.072–0.074	2
*PTPI-nigripes-A*	260–261	0.015–0.017	2

**Fig 1:**
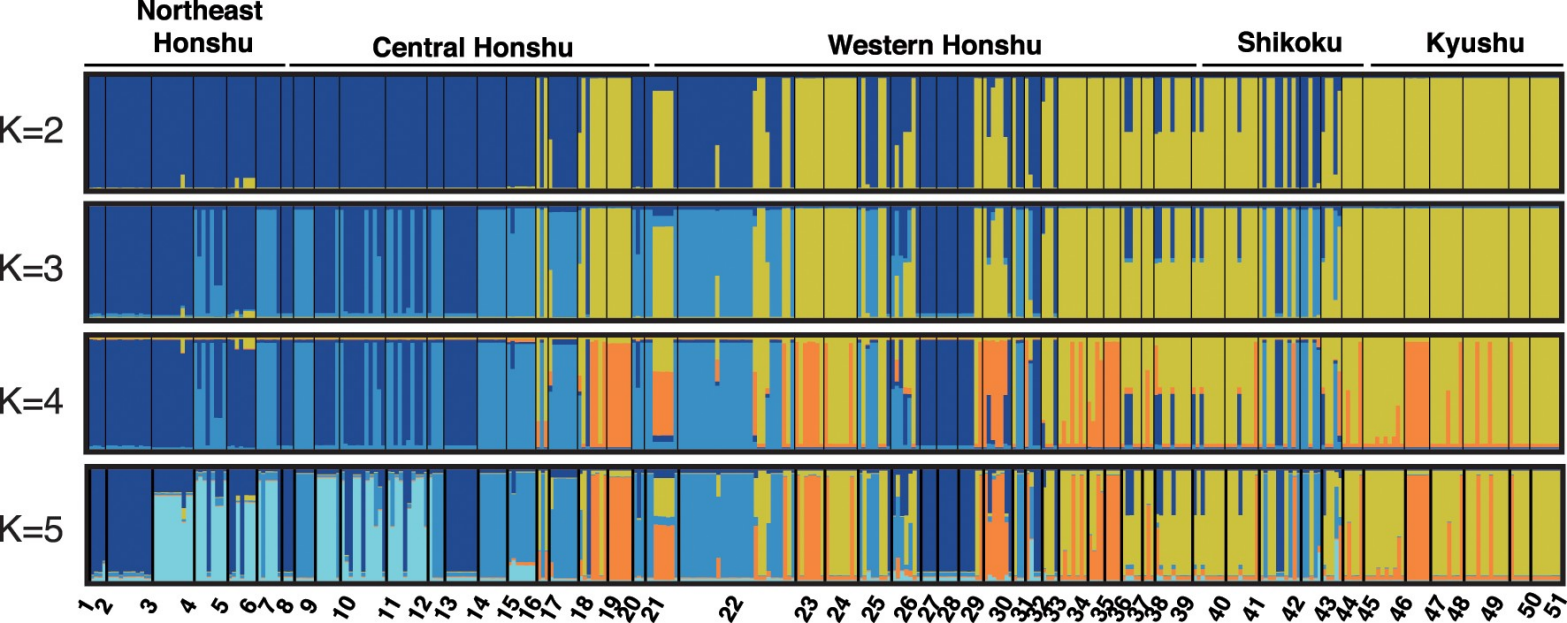
Results of InStruct analysis for full dataset. The proportion of the membership coefficient of 352 individuals in the 51 populations for each of the inferred clusters for K = 2–5 defined using Bayesian clustering in InStruct analysis. Each individual is shown as a column, and populations are separated from each other by a bold black line. Numerals at the bottom indicate population numbers.

**Fig 2:**
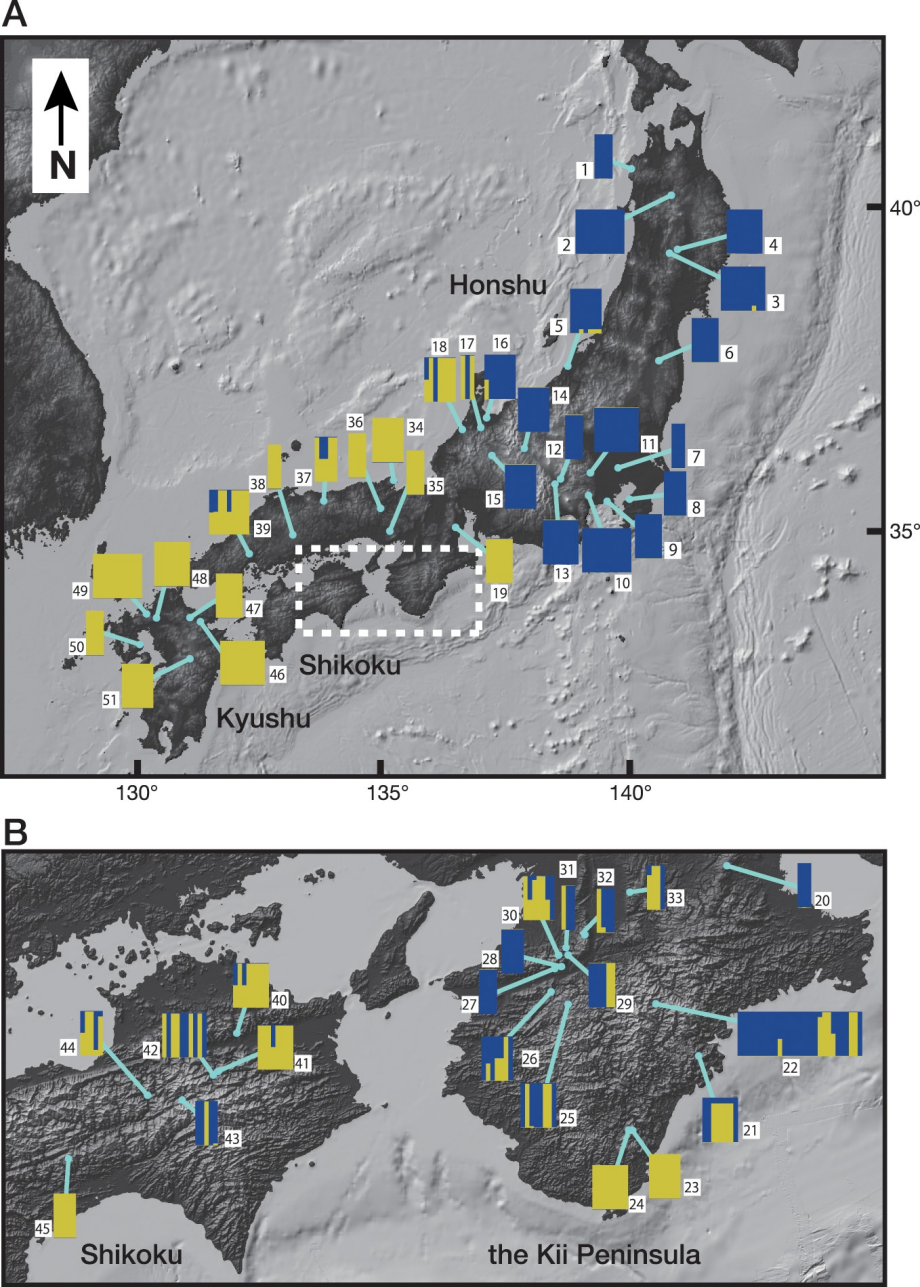
Distribution of each cluster at K = 2 throughout the Japanese archipelago. The distribution in the regions enclosed with a white dashed line in (A) is shown in (B). This map was drawn by GeoMapApp (http://www.geomapapp.org/) [53] / CC BY / CC BY (Ryan et al. [54]).

**Fig 3:**
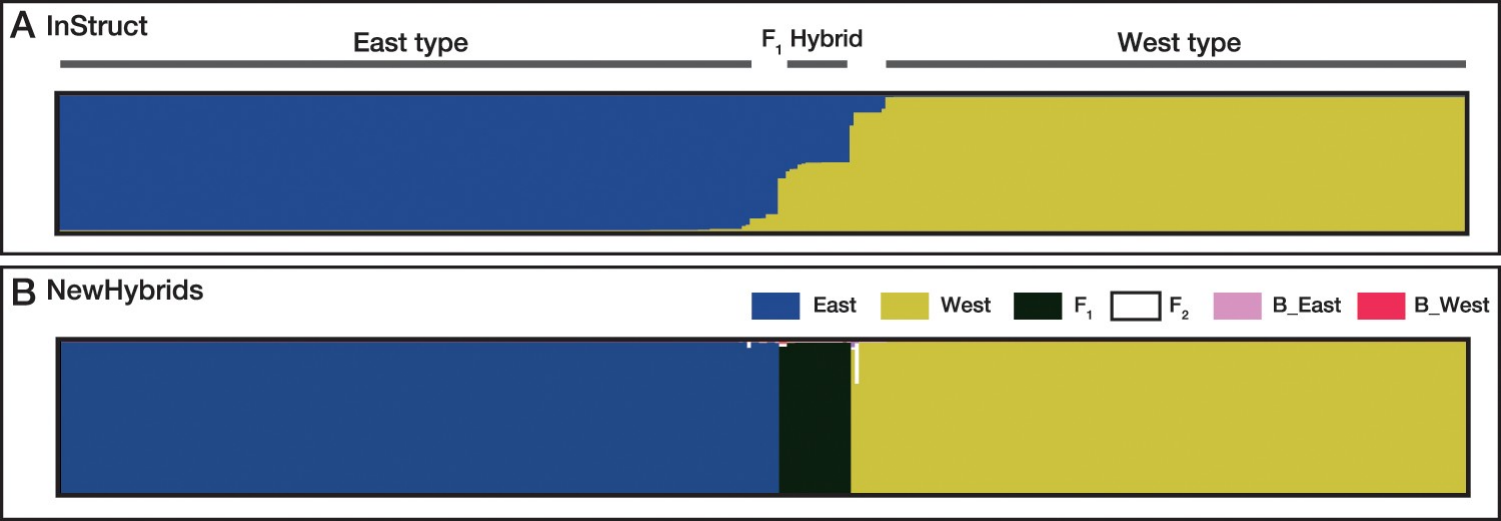
Identification and classification of non-admixed individuals (pure strains) and admixed individuals (hybrids) based on InStruct (A) and NewHybrid (B).

The InStruct results for full dataset and trimmed dataset indicated a clear population genetic structure among populations in *L*. *nigripes* (Figs 1 and 2 and S1 Fig). The Δ*K* method suggested that *K* = 2 was the optimal number of clusters for both datasets, although other methods proposed by Puechmaille [49], MedMeaK, MaxMeaK, MedMedK and MaxMedK indicated that K = 4 or 5 and K = 4 were the optimal number of clusters for the full dataset and trimmed dataset, respectively (S5 Table). At *K* = 2, one cluster (blue) included most individuals from north-eastern to central Honshu and some of the individuals from western Honshu and Shikoku, whereas the other (yellow) cluster comprised individuals from Shikoku and Kyushu and some of the individuals from central and western Honshu (Fig 1A). At K = 4 and 5, although each of two clusters at *K* = 2 were separated into additional clusters, the distinctness between the two clusters at *K* = 2 was consistently maintained (Fig 1A). Given that both of datasets, full dataset and trimmed dataset supported K = 2 in Δ*K* method, the result from Δ*K* method was robust against uneven sampling in this study. Thus, we used the result at K = 2 for subsequent analyses. The membership coefficients of the two clusters (*Q*-values) were plotted on the map (Fig 2). It was clear that *L*. *nigripes* showed a pattern of East-West genetic differentiation along the Japanese archipelago, although individuals of both clusters were commonly observed on the Kii Peninsula and on Shikoku Island (Fig 2B). Hereafter, we refer to the individuals belonging to the blue cluster as the East-type and those belonging to the yellow cluster as the West-type (Fig 2). The bar plot ordered by the *Q*-value at *K* = 2 indicated that admixed individuals (*Q*-values within the range of 5–95%) were relatively limited (34 individuals; 9.7%), despite the fact that the two clusters co-existed in the wide transitional zone (Figs 2B and 3). Among the admixed individuals, 16 individuals were interpreted as F_1_ because their genotypes (six MLGs) exhibit heterozygosity with the alleles derived from each of pure East- and West-types at all loci polymorphic among them (Fig 3A, Table 4). Their *Q*-values ranged from 0.476 to 0.552. The other individuals could be later-generation hybrids or backcross hybrids. The admixed individuals always co-existed with the East- and/or West-type within populations, and there was no case where a population consisted of only admixed individuals (Figs 1 and 2). On the other hand, NewHybrids assigned 18 individuals as F_1_ with high probability (> 0.9) (Fig 3B). The other individuals suggested to be admixed in the InStruct analysis were interpreted as the East-type or West-type, except for one individual (MLG43) that was assigned to F_2_ or backcross with a relatively higher probability than that observed for other individuals.

**Table 4:** Multi-locus genotypes of *Lepisorus nigripes* in this study.

	*PgiC*		*GapCp*		*pTPI*		Num.	Type
	*nigripes-T*	*nigripes-A*	*nigripes-T*	*nigripes-A*	*nigripes-T*	*nigripes-A*		
MLG1	**4_4**	**1_1**	**2_2**	**3_3**	**1_1**	**2_2**	**91**	West
MLG2	**2_2**	**1_1**	**1_1**	**2_2**	**1_1**	**1_1**	**55**	East
MLG3	**2_2**	**1_1**	**1_1**	**1_1**	**1_1**	**1_1**	**45**	East
MLG4	**1_1**	**1_1**	**1_1**	**2_2**	**1_1**	**1_1**	**13**	East
MLG5	**2_2**	**1_1**	**1_1**	**1_1**	**2_2**	**1_1**	**13**	East
MLG6	**1_1**	**1_1**	**1_1**	**1_1**	**1_1**	**1_1**	**12**	East
MLG7	**3_3**	**1_1**	**2_2**	**3_3**	**1_1**	**2_2**	**11**	West
MLG8	**6_6**	**1_1**	**2_2**	**3_3**	**1_1**	**2_2**	**10**	West
MLG9	**9_9**	**1_1**	**2_2**	**3_3**	**1_1**	**2_2**	**10**	West
MLG10*	**2_4**	**1_1**	**1_2**	**1_3**	**1_1**	**1_2**	**9**	F_1_
MLG11	**11_11**	**1_1**	**1_1**	**1_1**	**1_1**	**1_1**	**9**	East
MLG12	**2_2**	**1_1**	**2_2**	**3_3**	**1_1**	**2_2**	**7**	Recombinant
MLG13	**6_6**	**1_1**	**1_1**	**2_2**	**1_1**	**1_1**	**6**	East
MLG14	**12_12**	**1_1**	**2_2**	**3_3**	**1_1**	**2_2**	**6**	West
MLG15	**4_4**	**2_2**	**1_1**	**1_1**	**1_1**	**1_1**	**4**	Recombinant
MLG16*	**1_10**	**1_1**	**1_1**	**1_2**	**1_1**	**1_1**	**3**	East
MLG17	**4_4**	**1_1**	**3_3**	**3_3**	**1_1**	**2_2**	**3**	West
MLG18	**6_6**	**1_1**	**3_3**	**3_3**	**1_1**	**2_2**	**3**	West
MLG19	**7_7**	**1_1**	**1_1**	**2_2**	**1_1**	**1_1**	**3**	East
MLG20*	**4_5**	**1_1**	**2_2**	**3_3**	**1_1**	**2_2**	**3**	West
MLG21*	**2_2**	**1_1**	**1_1**	**1_2**	**1_1**	**1_1**	**2**	East
MLG22*	**2_2**	**1_1**	**1_2**	**2_3**	**1_1**	**1_2**	**2**	Recombinant
MLG23*	**2_4**	**1_1**	**1_2**	**2_3**	**1_1**	**1_2**	**2**	F_1_
MLG24*	**2_6**	**1_1**	**1_3**	**2_3**	**1_1**	**1_2**	**2**	F_1_
MLG25*	**3_4**	**1_1**	**2_2**	**3_3**	**1_1**	**2_2**	**2**	West
MLG26*	**1_2**	**1_1**	**1_1**	**1_2**	**1_1**	**1_1**	**1**	East
MLG27	**2_2**	**1_1**	**1_1**	**1_1**	**1_1**	**?_?**	**1**	East
MLG28	**2_2**	**1_1**	**1_1**	**?_?**	**?_?**	**1_1**	**1**	East
MLG29	**2_2**	**1_1**	**4_4**	**1_1**	**1_1**	**1_1**	**1**	East
MLG30	**2_2**	**1_1**	**?_?**	**?_?**	**1_1**	**1_1**	**1**	East
MLG31*	**2_4**	**1_1**	**1_1**	**2_2**	**1_1**	**1_1**	**1**	Recombinant
MLG32*	**2_4**	**1_1**	**1_3**	**1_3**	**1_2**	**1_2**	**1**	F_1_
MLG33*	**2_4**	**1_1**	**1_3**	**2_3**	**1_2**	**1_2**	**1**	F_1_
MLG34*	**2_6**	**1_1**	**1_1**	**1_2**	**1_1**	**1_1**	**1**	East
MLG35*	**2_7**	**1_1**	**1_1**	**1_1**	**1_1**	**1_1**	**1**	East
MLG36*	**2_9**	**1_1**	**1_2**	**1_3**	**1_1**	**1_2**	**1**	F_1_
MLG37*	**2_10**	**1_1**	**1_1**	**1_1**	**2_2**	**1_1**	**1**	East
MLG38	**3_3**	**1_1**	**3_3**	**?_?**	**1_1**	**2_2**	**1**	West
MLG39*	**3_4**	**1_1**	**3_3**	**3_3**	**1_1**	**2_2**	**1**	West
MLG40	**4_4**	**1_1**	**1_1**	**1_1**	**1_1**	**1_1**	**1**	Recombinant
MLG41	**4_4**	**1_1**	**1_1**	**2_2**	**1_1**	**1_1**	**1**	Recombinant
MLG42	**4_4**	**1_1**	**1_1**	**2_2**	**1_1**	**2_2**	**1**	Recombinant
MLG43	**4_4**	**1_1**	**2_2**	**1_1**	**1_1**	**2_2**	**1**	Recombinant
MLG44*	**6_6**	**1_1**	**2_3**	**3_3**	**1_1**	**1_2**	**1**	Recombinant
MLG45	**7_7**	**1_1**	**1_1**	**1_1**	**1_1**	**1_1**	**1**	East
MLG46	**8_8**	**1_1**	**2_2**	**3_3**	**1_1**	**2_2**	**1**	West
MLG47	**9_9**	**1_1**	**1_1**	**2_2**	**1_1**	**1_1**	**1**	Recombinant
MLG48	**10_10**	**1_1**	**1_1**	**1_1**	**2_2**	**1_1**	**1**	East
MLG49	**?_?**	**1_1**	**2_2**	**3_3**	**1_1**	**2_2**	**1**	West
MLG50*	**3_6**	**1_1**	**2_2**	**3_3**	**1_1**	**2_2**	**1**	West
MLG51	**5_5**	**1_1**	**2_2**	**3_3**	**1_1**	**2_2**	**1**	West

Different number in each locus indicates different allele. ‘?’ missing data. In each locus, two alleles are combined with ‘_’ to express genotype in each locus. MLG names with asterisk indicate MLGs with at least one heterozygous locus. In type column, each MLG was classified into East, West, F_1_ and Recombinant based on the result in InStruct (see Materials and Methods and Result in the article).

**Fig 4:**
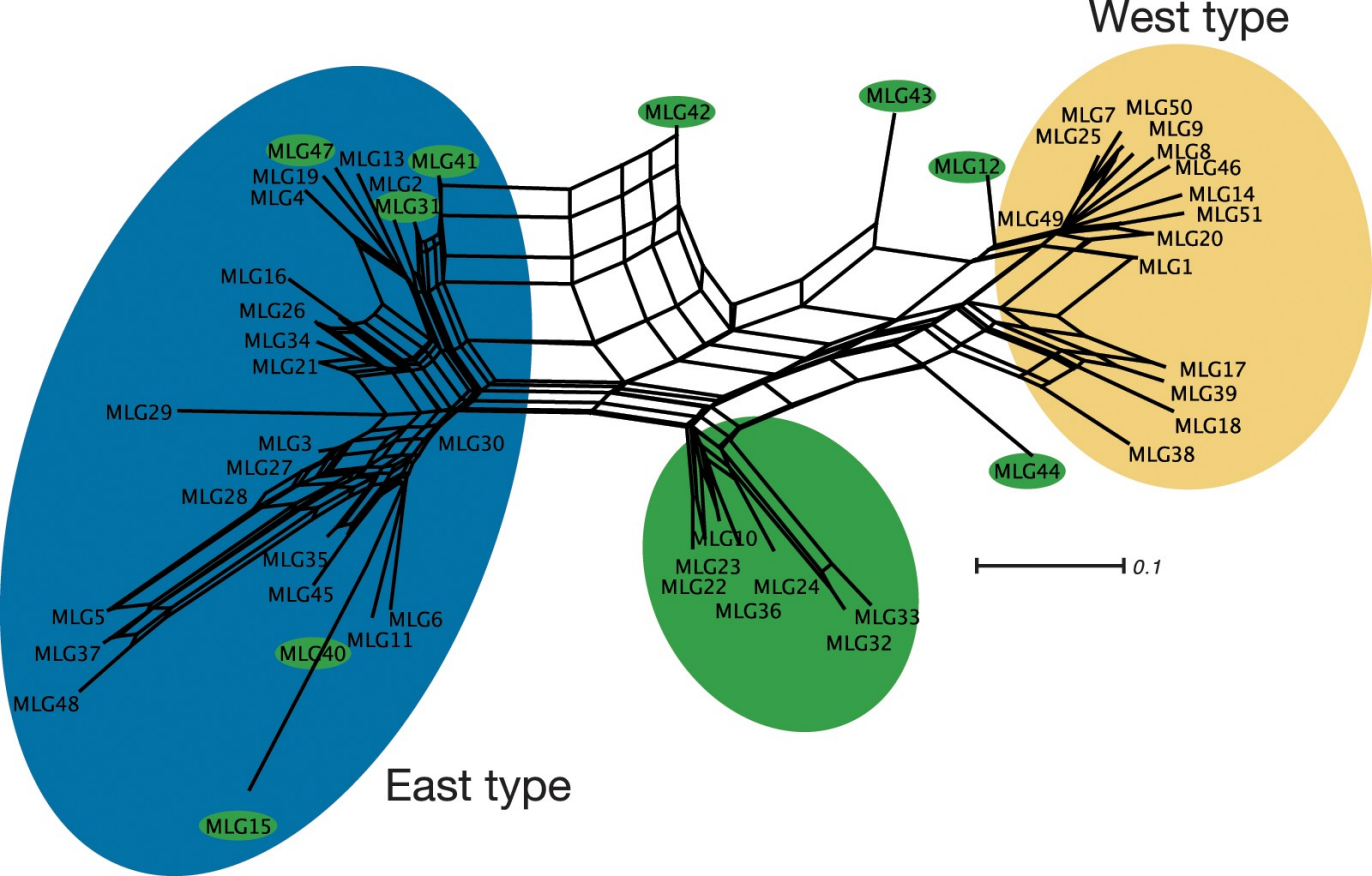
Neighbor-net analysis for multilocus genotypes. Blue-, yellow- and green-coloured MLGs indicate MLGs of the East-type, the West-type, and hybrids or recombinants between the East- and West-types.

To assess the genetic relationship among MLGs, neighbor-net was constructed (Fig 4). The MLGs of the East-type and those of the West-type were separated into two different groups, consistent with the InStruct result at K = 2. The six MLGs estimated to be F_1_ (Fig 3) formed a cluster with MLG22 in the middle of the branches connecting the East and West types. The other admixed MLGs were located discretely between the two groups or were nested in the group of the East-type.

The proportions of each MLG in each geographic region are shown in S2 Fig. Northern Honshu (the Tohoku District) and central Honshu (the Kanto and Chubu Districts) showed a high diversity of MLGs from the East-type and harboured MLGs unique to each of the two regions. By contrast, a high diversity of MLGs and unique MLGs from the West-type was observed in Western Honshu (the Kinki region and the Chugoku District), Shikoku and Kyushu. Although the East- and West-types co-existed and produced F_1_ hybrids (dark green in S2 Fig) in western Honshu and Shikoku, the frequencies of recombinant MLGs (light green) were very low, except for MLG12.

Because both the Bayesian clustering (InStruct) and agglomerate clustering (neighbor-net) methods revealed two genetically distinct entities, the East- and West-types in *L*. *nigripes*, we treated samples of the East- and West-types as belonging to different populations in the subsequent population genetic analyses when they were collected from the same location. The AMOVA results revealed that most of the genetic variation in *L*. *nigripes* was distributed between the East- and West-types: *F*rt = 0.759 (Table 5). Within each type, genetic differentiation among geographic regions (*F*rt) was not significant. However, genetic differentiation among populations (*F*st) was high for both types: 0.652 (East-type) and 0.399 (West-type). Allelic richness (*A*r) and inbreeding coefficient (*F*) values for each population are presented in Table 1. The *F* values ranged from 0.000 to 1.000 with a mean of 0.730 in the East-type populations. The West-type populations also showed high levels of *F* values, ranging from 0.250 to 1.000 with a mean of 0.824. High levels of inbreeding were also suggested by Instruct (*F*is = 0.900 and 0.940) and AMOVA (*F*is = 0.767 and 0.821).

**Table 5:** AMOVA results comparing the genetic variation between the East- and West-types and among regions for *Lepisorus nigripes*.

	df	Variance component		Percent variation	Φ-statistics	
Nuclear DNA variation						
**Between East- and West-types**						
Between types	1	1.567		76%	*F*rt	**0.759**
Among populations within types	22	0.297		14%	*F*sr	**0.597**
Among individuals	175	0.157		8%	*F*st	**0.903**
Within individuals	199	0.043		2%	*F*is	**0.786**
**Within the East-type**						
Among regions ^#^	2	0.000		0%	*F*rt	-0.066
Among populations within regions	9	0.483		67%	*F*sr	**0.674**
Among individuals	98	0.179		25%	*F*st	**0.652**
Within individuals	110	0.055		8%	*F*is	**0.767**
**Within the West-type**						
Among regions ^$^	2	0.000		0%	*F*rt	-0.044
Among populations within regions	9	0.116		42%	*F*sr	**0.424**
Among individuals	77	0.129		47%	*F*st	**0.399**
Within individuals	89	0.028		10%	*F*is	**0.821**
Chloroplast DNA variation						
**Between East- and West-types**						
Between types	1	0.033	12%		*F*pt	**0.120**
Within types	60	0.238	88%			

Φ-statistics values that deviate significantly from zero are shown by bold characters.

#, three regions, northern, central, and western Honshu, were considered.

$, three regions, Western Honshu, Shikoku, and Kyushu Islands, were considered.

### Phylogenetic analysis

The aligned sequences of nuclear genes were 650 bp for *PgiC*, 690 bp for *GapCp* and 281 bp for *pTPI*. GTR+G was selected as the best fitting model for *PgiC*, and HKY+G for *GapCp* and *pTPI*. We obtained a multiple sequence alignment of 1009 bp including 14 gaps for the chloroplast *rps4* –*trnS* region. GTR+G was selected as the best-fitting model.

**Fig 5:**
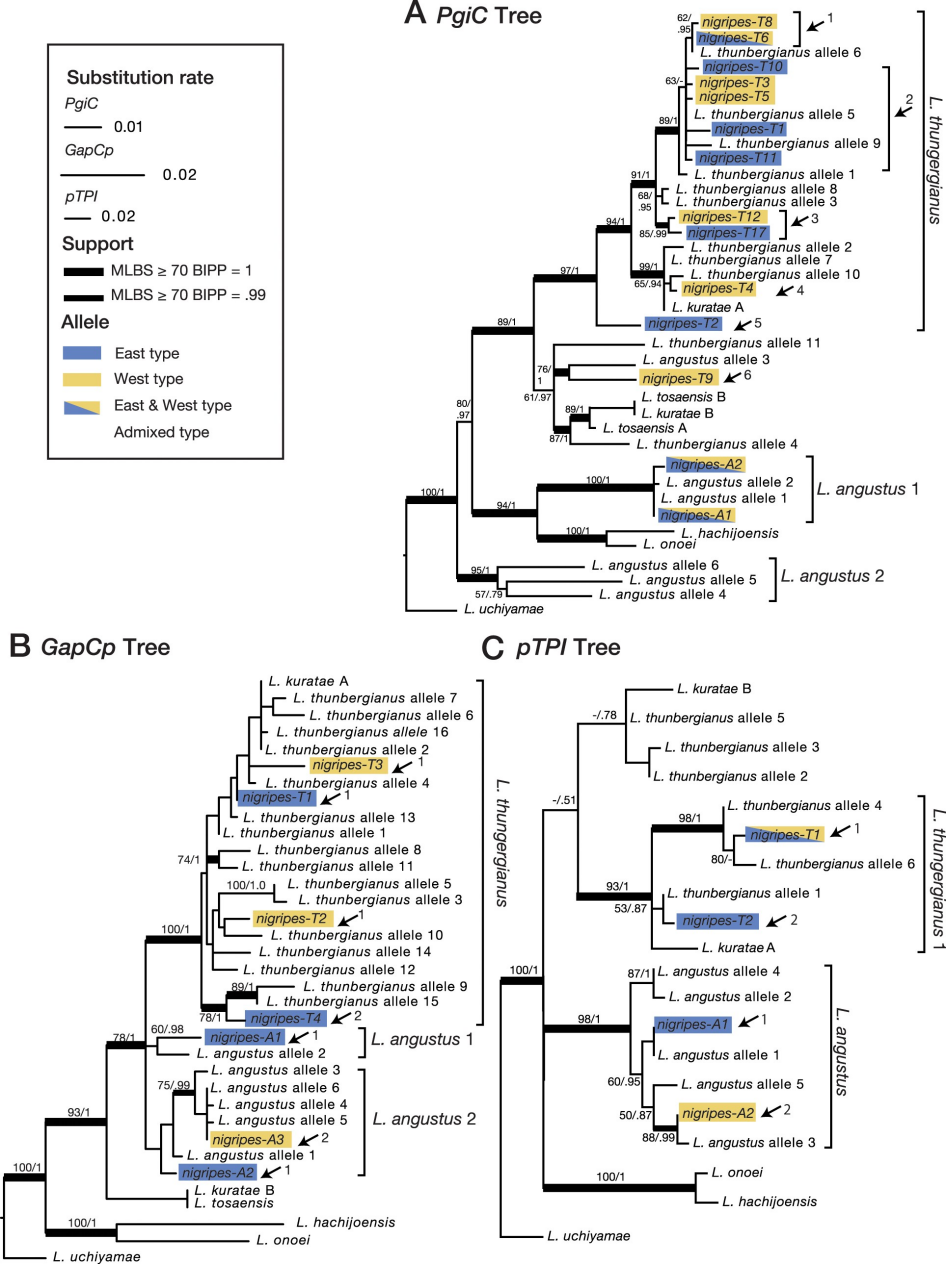
ML phylogenetic trees of nuclear genes, *PgiC* (A), *GapCp* (B) and *pTPI* (C). The thickest lines indicate the strongest support (MLBS > = 70, BIPP = 1.0), moderately thick lines indicate moderate support (MLBS > = 70, BIPP = 0.99), and thin lines indicate weak support (MLBS < 70 or BIPP < 0.99). Support values are given as MLBS / BIPP. *nigripes-T* and *nigripes-A* indicate the alleles in *Lepisorus nigripes* derived from *L*. *thunbergianus* and *L*. *angustus*, respectively. Blue and yellow colours indicate alleles from the East- and West-types, respectively. Arrows indicate the different inferred origins.

The ML tree of *PgiC* (Fig 5A) showed four highly supported clades (*L*. *thunbergianus* clade, *L*. *angustus*_1 clade, *L*. *angustus*_2 clade, and a clade of *L*. *hachijoensis* + *L*. *onoei*), as well as a weakly supported sister clade to the *L*. *thunbergianus* clade. Most of the sequences from *L*. *thunbergianus* and those at the “*nigripes-T*” locus in *L*. *nigripes* (12 sequences) were included in the *L*. *thunbergianus* clade. The sequences from *L*. *angustus* occurred mainly in two separate clades: *L*. *angustus*_1 and *L*. *angustus*_2. Two sequences at the ‘*nigripes-A*’ locus in *L*. *nigripes* (*nigripes-A1* and *nigripes-A2*) were included in the *L*. *angustus*_1 clade. Two sequences from *L*. *thunbergianus* (*L*. *thunbergianus* alleles 4 and 11), a sequence from the “*nigripes-T*” locus in *L*. *nigripes* (*nigripes-T9*), and a sequence from *L*. *angustus* (*L*. *angustus* allele 3) were included in the weakly supported clade that was sister to the *L*. *thunbergianus* clade, together with the sequences from *L*. *tosaensis* and *L*. *kuratae*.

*GapCp* tree (Fig 5B) comprised four clades and a unique sequence (*L*. *tosaensis* / *L*. *kuratae*). All sequences from *L*. *thunbergianus* and four sequences at the “*nigripes-T*” locus in *L*. *nigripes* were included in the *L*. *thunbergianus* clade. Within this clade, most of the subclades were not supported. The sequences from *L*. *angustus* and those at the ‘*nigripes-A*’ locus in *L*. *nigripes* were included in two clades: *L*. *angustus*_1 clade and *L*. *angustus*_2 clade. *nigripes-A1* of *L*. *nigripes* was included in the *L*. *angustus* 1 clade, together with *L*. *angustus* allele 2. In the *L*. *angustus*_2 clade, *nigripes-T3* clustered with *L*. *angustus* alleles 4, 5 and 6 with relatively high support (75/0.99).

The ML tree of *pTPI* (Fig 5C) displayed three highly supported clades and an unsupported clade of *L*. *thunbergianus* alleles and *L*. *kuratae* B. *nigripes-T1* and *nigripes-T2* from *L*. *nigripes* were included in the *L*. *thunbergianus*_1 clade and clustered with *L*. *thunbergianus* alleles 4 and 6 and with *L*. *thunbergianus* allele 1, respectively. All of the sequences from *L*. *angustus* and those at the ‘*nigripes-A*’ locus in *L*. *nigripes* formed a highly supported clade (98/1.0). In this clade, *nigripes-A1* from *L*. *nigripes* was identical to *L*. *angustus* allele 1, and *nigripes-A2* was grouped with *L*. *angustus* allele 3.

With respect to the cpDNA tree, the ML and BI analyses yielded the same topology (S3 Fig). All of the haplotypes of *L*. *nigripes* and those of *L*. *thunbergianus* were included in the same clade (MLBS = 99, BIPP = 1.0). Hap_C was the most frequent haplotype and was shared by both East- and West-type individuals from 35 populations throughout the Japanese archipelago. Hap_D1 and Hap_D2 were unique to the East-type, and Hap_B was unique to the West-type.

### Fertility of spores in F_1_ hybrids between East- and West-types

**Fig 6:**
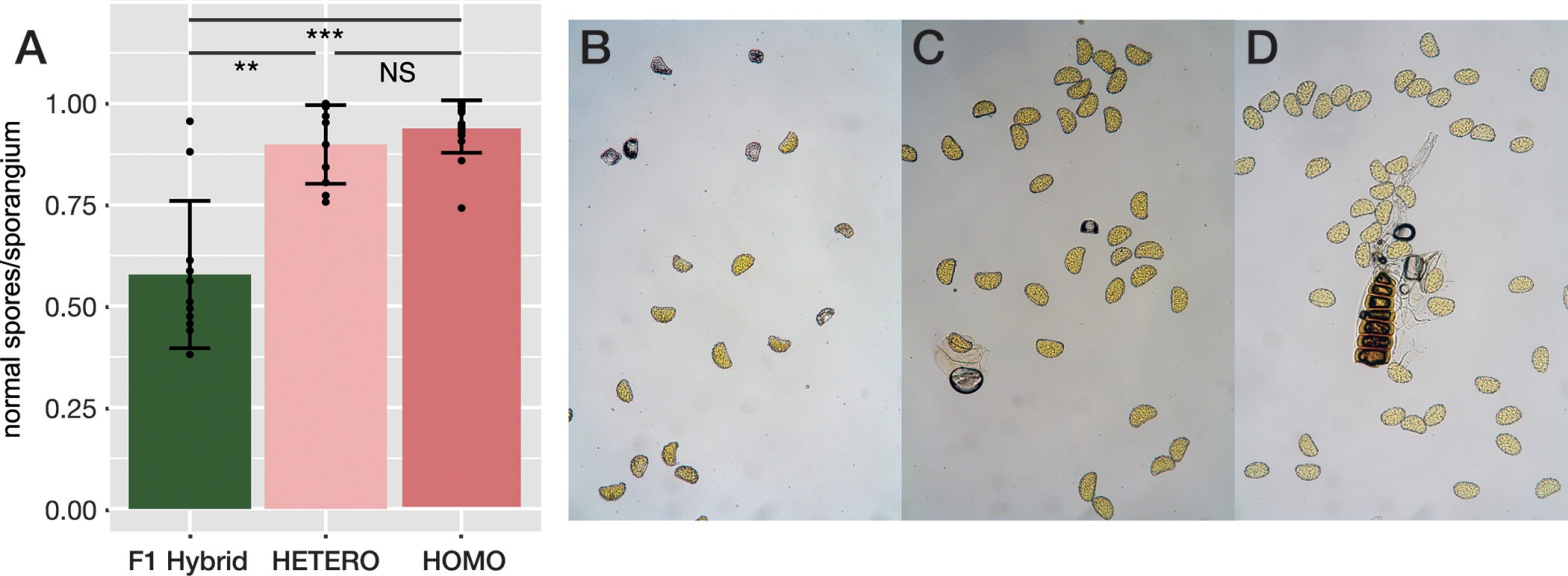
The level of fertility among three categories, F_1_ hybrids, heterozygous individuals within each type and homozygous individuals within each type. The proportion of normal spores in F_1_ hybrids between types (F_1_ hybrid in the figure, left), heterozygous individuals within each type (HETERO in the figure, centre), and homozygous individuals within each type (HOMO in the figure, right) (A), and photographs of spore morphologies in each category: (B) an F_1_ hybrid (sample ID 26–4) between types, (C) an heterozygous individual (sample ID 15–2) of the East-type, and (D) an homozygous individual (sample ID 15–1) of the East-type. In the graph (A), each point in the bar indicates an individual data point; error bars represent SE (±). Asterisks represent differences at confidence levels of ** *p* < 0.005 and *** *p* < 0.001.

The mean proportions of normal spores per sporangium in each of the putative F_1_ hybrids between the East and West types from seven populations (Populations 17, 18, 22, 26, 30, 39 and 40) ranged from 0.381 to 0.956 with an average value of 0.59. Two individuals from Population 39 showed exceptionally high values of 0.881 and 0.956, and the other F_1_ individuals had values around 0.500 (Fig 6). With respect to non-admixed individuals, regardless of homozygosity or heterozygosity, mean proportions of normal spores ranged from 0.75 to 1.0, and average values exceeded 0.9. The proportion of normal spores of F_1_ hybrids was significantly lower than that of non-admixed homozygous individuals (P < 0.001) and heterozygous individuals (P < 0.01).

## Discussion

### Genetic differentiation between East and West Japan

In Bayesian clustering analysis, Δ*K* method selected K = 2 as optical number of clusters for full dataset and trimmed dataset (S5 Table), which suggested the result of Δ*K* method in this study was robust against uneven population sampling. At K = 2, the samples of *Lepisorus nigripes* were separated into two clusters, East- and West-types (Figs 1 and 2 and S1 Fig). The East-type is distributed mainly in northern and central Honshu, while the West-type is distributed mostly in western Honshu, Shikoku, and Kyushu. This obvious geographical and genetic distinction suggested that the populations in the East- and West-types have been geographically isolated from each other. Although the East- and West-types often co-exist on the Kii Peninsula and on Shikoku Island (Figs 1 and 2B), only four East-type MLGs (MLG2, 3, 21 and 24) out of 17 pure East-type MLGs were found there despite that 8 of 13 non-admixed West-type MLGs (62%) were found (Table 4, S2 Fig and S4 Table) and the geographic distribution of *A*r values showed the reduction of allelic richness of East-type in Western Honshu and Shikoku island (S3 Fig). These results imply that the coexistence of East- and West-types on the Kii Peninsula and on Shikoku Island could be caused by recent range expansion of both of types. Other methods to determine optimal number of clusters, MedMeaK, MaxMeaK, MedMedK and MaxMedK selected K = 4 and 5 as the optimal number (S5 Table). However, the separations were the subdivision of each of cluster at K = 2, East-and West-types (Fig 1). Additionally, most of admixed individuals between clusters inside East- or West-type at K = 4 and 5 were heterozygous individuals within each type, whose fertilities were same as those in non-admixed individuals (Fig 6 and S4 Table), as discussed later. Thus, it was concluded that the distinction at K = 2 is more biologically significant. The genetic differentiation between the East- and West-types at K = 2 was also supported by the neighbor-net analysis (Fig 4), the sharing of a few alleles in nuclear phylogenetic trees (Fig 5), and the high *F*rt values (0.759) in Φ-statistics (Table 5).

An East-West pattern of intraspecific genetic differentiation in the Japanese archipelago has often been reported in phylogeographic studies on woody angiosperm species [34,35,73–76]. The present study is the first case of the East-West differentiation pattern in fern species in the Japanese archipelago. The scarcity of this differentiation pattern in ferns may reflect the high dispersal ability of ferns that have small spores as propagules [77], or merely that few phylogenetic studies have been conducted on Japanese ferns so far. The phylogeographic studies on Japanese plants concluded that such a geographic genetic structure would be caused by climatic changes during repeated glacial periods in the Quaternary period. During the last glacial maximum (LGM), the climate in the Japanese archipelago was much colder than at present. Evergreen and deciduous forests have been considered to have reduced their population sizes and have been restricted to small refugia. The East-West differentiation pattern is considered to have been formed by the past geographical isolation between eastern and western refugia during the LGM, followed by range expansion. We suggest that the geographical distinction between the East- and West-types of *L*. *nigripes* was also generated through geographical isolation between refugia formed during the glacial period(s) in the Quaternary. It is likely that epiphytic *L*. *nigripes* shifted its distribution together with its host tree species. We present two possible scenarios depending on the different timing of allopolyploidization events. (1) Ancestors of the East- and West-types originated before the last glacial period. With the arrival of the last glacial period, their geographical distribution range decreased, and their populations were restricted to eastern and western refugia. The geographic isolation during the last glacial period eventually resulted in the formation of genetically distinct groups, the East and West types. If (1) did not occur, (2) East- and West-types of *L*. *nigripes* originated independently in geographically isolated refugia in Eastern and Western Japan, respectively, during the last glacial period. The genetic difference between the two may reflect that they originated in different locations and from different parental source populations.

AMOVA analysis revealed that the levels of genetic differentiation between East- and West-types is Frt = 0.759 and the differentiation is the primary source of whole variation observed in *L*. *nigripes* (Table 5). Some studies also detected East-West differentiation in other Japanese plant species and estimated the levels of genetic differentiation between eastern and western groups using several nuclear markers such as AFLP, CAPS and SSR, showing Fct = 0.014 for *Castanopsis sieboldii* (Makino) Hatus. ex T.Yamaz. et Mashiba with EST-SSRs [78], Fst = 0.0396 in *Chamaecyparis obtuse* (Siebold & Zucc.) Endl. with CAPS [79], Fct = 0.23 in *Viola eizanensis* Makino (Makino) with AFLP [80] and Fct = 0.35 in *Cardamine scutata* Thunb. with AFLP [81]. Importantly, AMOVA analysis conducted in these studies showed that genetic differentiation between the groups was not the primary source of genetic variation. Comparing with East-West differentiation in other plants that experienced population isolation between eastern and western Japan during LGM, the genetic differentiation between East- and West-types in *L*. *nigripes* showed much higher level of differentiation and had stronger effect on whole genetic variation in this species, which suggested that the genetic differentiation in *L*. *nigripes* cannot be explained only by bottleneck effect in geographical isolation of populations, that was cause for East-West differentiation in other plants. In addition, it should be noted that the East- and West-types shared few or no alleles at the nuclear loci examined: 1/12 and 1/2 for *PgiC*, 0/4 and 0/3 for *GapCp*, and 1/2 and 0/2 for *pTPI* (Fig 5). A high level of genetic differentiation would be unlikely if these types diverged only through genetic drift during the last glacier period, i.e., less than one hundred thousand years ago. Therefore, the scenario 2 is more plausible and it is likely that the genetic distinctness between the East- and West-types of *L*. *nigripes* reflects the differential introduction of parental allelic variants to polyploids in their respective place of origin, i.e., one type of founder effect. Independent origin of East- and West-types will also be supported by phylogenetic analyses (see the discussion below).

Although *L*. *nigripes* is widely distributed in both cool and warm temperate regions [28], its parental species, *L*. *thunbergianus* and *L*. *angustus*, are restricted to warm temperate and cool temperate regions in Japan, respectively [33] Thus, it is likely that the geographical distribution areas of the parental species were strongly influenced by climatic oscillations during the Quaternary Era, together with the evergreen and/or deciduous forest trees on which they grow. Aoki et al. [74] revealed genetic differentiation in *Curculio sikkemensis* (Heller) between the north-eastern and southwestern parts of the mainland of Japan; this is a generalist acorn weevil of Fagaceae plants inhabiting deciduous forest and evergreen forest. Because no significant genetic differentiation of the weevil was observed between vegetation types of its utilized host plant species, Aoki et al. [74] concluded that deciduous and evergreen forests might have survived together or adjacent to each other during glacial periods. Qi et al. [82] estimated the potential geographic distribution of a Japanese deciduous cool temperate tree, *Cercidiphyllum japonicum* Siebold et Zucc. ex Hoffm. et Schult., during the LGM based on ecological niche modelling. They found that *C*. *japonicum* exhibited a southward range shift during the LGM and occupied areas where warm temperate forests develop at present. In this light, it is likely that *L*. *thunbergianus* and *L*. *angustus* also survived in the same or adjacent regions during glacial periods. Therefore, we can anticipate more opportunities for interspecific hybridization that is essential for producing *L*. *nigripes* during glacial periods compared with current and past warm interglacial periods.

### Independent multiple origins

Nuclear DNA phylogenies revealed that *L*. *nigripes* recurrently originated from independent polyploidization events. In *pTPI* trees (Fig 5C), *nigripes-A1* fixed in the East type of *L*. *nigripes* was identical to *L*. *angustus* allele 1, while *nigripes-A2* fixed in the West-type clustered with *L*. *angustus* allele 3 with high support. This result indicates that *nigripes-A1* and *nigripes-A2* are the sequences derived from different individuals of *L*. *angustus*. Such a derivation pattern was observed also in the *GapCp* tree (Fig 5B). *nigripes-A3* fixed in the West-type formed a clade together with *L*. *angustus* alleles 4, 5 and 6, whereas *nigripes-A1* and *nigripes-A2*, specific to the East-type, were not included in the clade, suggesting that *nigripes-A3* in the West-type and *nigripes-A1* and *nigripes-A2* in the East-type emerged from different origins. The result that each allele in *GapCp*- *nigripes-A* and *pTPI*-*nigripes-A* from the East- and West-types emerged independently from different parents supports scenario 2 in the above section, i.e., that the East- and West-types originated independently.

Alternatively, the possession of distinct alleles between East- and West-types also can be explained by two different mechanisms. One is that an ancestral allopolyploid with heterozygosity originated from single polyploidization and each allele was fixed in each of East- and West-types throughout gametophytic selfing. Another is introgression between *L*. *nigripes* and parental diploid species. Firstly, an ancestral allopolyploid with heterozygosity from single origin requires a fusion of two unreduced gametes with heterozygosity from two different diploid species. Given that gametophyte of *Lepisorus* is cordate-thalloid gametophyte that be known as short-live[83,84], the probability that unreduced spores were simultaneously produced from both parental species interbreeding with each other, is extremely low. It is rather much more likely that *L*. *nigripes* recurrently originated from single unreduced gametophytes produced in diploid hybrid species throughout gametophytic selfing, the ability unique to ferns. Additionally, two independent loci, *pTPI* in *L*. *angustus* subgenome and *GapCp* in *L*. *angustus* subgenome supported the pattern that can be interpreted as independent origins of East- and West-types. It is highly unlikely that the fixation of different alleles between the types in the two independent loci occurred throughout single origin. Secondly, interploidal introgression commonly occurs via triploid bridge in angiosperms. However, such a triploid bridge is unlikely in the case of *L*. *nigripes*, because triploid hybrid between *L*. *nigripes* and parental species has highly withered sporangia that cannot produce any spores and the evidence of introgression between two species with different ploidal level has never been reported so far in ferns. Therefore, independent origin is the most plausible explanation for the pattern observed in *pTPI* and *GapCp* phylogenies.

The *PgiC* tree provides evidences for additional independent origins. *nigripes-T8* and *nigripes-T6* formed a clade together with *L*. *thunbergianus* allele 6 (arrow 1 in Fig 5A), whereas *nigripes-T1*, *3*, *5*, *10*, and *11* branched from the base of the clade (arrow 2), suggesting at least two independent acquisition events of diploid alleles. *nigripes-T7* and *nigripes-T12* formed a clade that diverged from a more basal position (arrow 3), which reflected the other origin. *nigripes-T4* was included in the clade that consisted of *L*. *thunbergianus* alleles 2, 7, 10, and the sequence of *L*. *kuratae* homoeologous loci A (arrow 4). *nigripes-T2* was a sister clade to all of the other sequences of the *L*. *thunbergianus* clade (arrow 5). Thus, *nigripes-T4* and *nigripes-T2* also have independent origins. Lastly, although *nigripes-T9* was the sequence from the “*nigripes-T*” locus of *L*. *nigripes*, this sequence was sister to *L*. *angustus* allele 3. Because the genetic distance between *nigripes-T9* and *L*. *angustus* allele 3 was relatively high and the sequences of *L*. *thunbergianus* were also nested and polyphyletic in this clade, we interpreted this sister relationship to be the result of incomplete lineage sorting, not as a result of *nigripes-T9* having been derived from *L*. *angustus* as opposed to *L*. *thunbergianus*. Therefore, *nigripes-T9* might have independently derived from *L*. *thunbergianus*. According to scenario 2, if we assume that the East- and West-types originated independently in different refugia, the East- and West-types had at least four (arrows 1, 2, 3, and 5) and five (arrows 1, 2, 3, 4, and 6) origins, respectively, based on the result of the *PgiC* tree. Multiple origins have been reported among several polyploid taxa in ferns: *Asplenium ceterach* has at least six origins [85], *A*. *cimmerioru*m has at least two origins, *A*. *gracillimum* has at least four origins [24], *A*. *majoricu*m has at least four origins [86], *Astrolepis integerrim*a has at least ten origins [87], and *Polypodium hesperiu*m has at least two origins [37]. It was suggested that the East- and West-types of *L*. *nigripes* originated recurrently approximately the same number of times as allopolyploid ferns examined in previous studies.

### Mating system of *Lepisorus nigripes*

Populations of *Lepisorus nigripes* exhibited high values of the inbreeding coefficient (*F*): a mean of 0.730 for the East-type populations and 0.824 for the West-type ones (Table 1). This level of inbreeding coefficient is comparable for the values in the species categorized as ‘exclusively inbreeding’ [88]. Masuyama et al. [46] performed gametophytic selfing tests for *L*. *nigripes* (treated as tetraploid *L*. *thunbergianus* in their study) and *L*. *thunbergianus* (treated as diploid *L*. *thunbergianus* in their study) individuals. In their test, 50 gametophytes per sporophyte were isolated and forced to undergo gametophytic selfing. In *L*. *thunbergianus*, only 0–16% of gametophytes produced sporophytes and some of the sporophytes were small and irregularly shaped. On the other hand, in *L*. *nigripes*, 98–100% produced sporophytes with a normal shape. Based on their results, the authors concluded that *L*. *nigripes* has high gametophytic selfing potential, which is consistent with our results. We found that populations of *L*. *nigripes* were characterized by low levels of genetic variation within the populations; ten out of 51 populations comprised only one MLG (Table 1). Gametophytic selfing enables ferns to colonize a new open habitat by a single spore [89,90], and it is expected that the population established through this process is initially genetically monomorphic [91]. The existence of such single-MLG populations observed in this study and the relatively high levels of *F*st values among populations (Table 5) could be evidence that gametophytic selfing frequently occurs in *L*. *nigripes* under natural conditions.

Although our understanding of the genetic, epigenetic, and genomic consequences of polyploid evolution in plants has greatly improved during the last two decades, how new polyploids establish and then persist in natural populations remains poorly understood [8]. Fowler and Levin [92] examined factors critical to the establishment of a new allopolyploid population based on a simulation model and suggested that a higher level of self-fertilization of polyploids increases the success rate because it mitigates the minority disadvantage [14] of the newly formed polyploids. Masuyama and Watano [93] suggested that gametophytic selfing might occur more frequently in polyploid than in diploid species. The contrasting mating systems of tetraploid *L*. *nigripes* and diploid *L*. *thunbergianus* [46] were adopted in the paper as a representative case. If evolution of selfing were triggered by the reduction of inbreeding depression in polyploids as suggested by Lande and Schemske [94], we could expect a substantial increase in selfing ability, even in a nascent polyploid. The idea that the mating system shift caused by polyploidization would assist the initial establishment of polyploids should be evaluated in future studies.

### Initial stage of reproductive isolation between the East- and West-types

InStruct revealed that admixed individuals between the East- and West-types were relatively limited despite their coexistence across wide areas (Figs 2 and 3). We found 36 admixed individuals among the 352 samples examined. 16 of them were interpreted as F_1_ hybrids, and 20 individuals were recombinants (Fig 3 and Table 4). In western Honshu and Shikoku, where the East- and West-types frequently co-exist, 15 recombinant individuals were found. The remaining five individuals with MLG15 and 40 were observed in northern Honshu, where the West-types were not found (Figs 1, 2 and 4). It is possible that MLG15 and 40 might not be recombinant genotypes, but rather MLGs unique to northern Honshu as suggested by neighbour-net analysis (Fig 4). Moreover, NewHybrids (Fig 3B) assigned 18 individuals as F_1_ hybrids with high probabilities, and only one individual showed a higher probability for F_2_ or backcrossing than that observed for the pure strain. The paucity of recombinants between the East- and West-types observed in this study suggested the presence of reproductive isolation between the two types. As *L*. *nigripes* is a predominantly selfing species, this mating system seems to have contributed towards the reduction of gene flow between the East- and West-types. However, it is unlikely that selfing alone could maintain the genetic distinctness across wide sympatric regions. Even in predominantly selfing species, occasional mating between selfing strains can occur, generating recombinant genotypes [95]. For example, although particular MLGs were maintained within each population in the predominantly selfing fern *Sceptridium ternatum* (Thunb.) Lyon, these MLGs were not maintained across populations [96]. Together, these findings suggest that intrinsic reproductive isolation is likely responsible for the genetic distinctness between the East- and West-types of *L*. *nigripes*. As shown in Fig 6, most of the F_1_ hybrids between the East- and West-types showed significant reductions in fertility compared to the spore fertility of non-admixed individuals. The mean proportion of normal spores among F_1_ hybrids was 0.59. Although two individual F_1_ hybrids of POP39 showed a high proportion of normal spores, a reduction in the fertility of F_1_ hybrids was widely observed across six populations from central Honshu, western Honshu, and Shikoku. This suggests that the reductions in spore fertility were not caused by external factors in each population, but rather by intrinsic factors of hybrid individuals.

Artificial crossing experiments between cryptic species that were detected by genetic markers in ferns were conducted in previous studies [71,97,98]. In the *Ceratopteris thallicroides* species complex, Masuyama et al. [71] showed that the germination rates in F_1_ hybrids from intraspecific crossing between different geographic sources were as high as 50%, while those from interspecific crossing were nearly 0%. They also calculated the frequency of abortive spores in each crossing, revealing frequency ranges between 0.0% and 18.6% for intraspecific crossing and between 64.6% and 91.0% for interspecific crossing. In the present study, the frequencies of abortive spores in F_1_ hybrids between the East- and West-types (38.7–61.9% except for the hybrids in POP39) were lower than those of interspecific hybrids in *Ceratopteris*. Additionally, because recombinant genotypes were found in the sympatric regions, we concluded that the intrinsic reproductive isolation between the East- and West-types would be incomplete. During the initial stage of divergence, the loci responsible for isolation can be polymorphic among individuals. As a result, the degree of incompatibility is expected to vary among crossing pairs [99]. Our observation that F_1_ hybrids in POP39 showed high proportions of normal spores might indicate variability in reproductive isolation between the East- and West-types.

## Conclusions

It remains unclear whether lineages with independent origins interbreed [8,9,22]. Symonds et al. [23] hypothesized the outcomes of multiple origins in polyploid species ranging from a single homogenous species to multiple genetically distinct lineages that ultimately form cryptic species, according to genetic variations contributed by parental species and the degree of gene flow among independent origins.

In this study, we detected two genetically distinct lineages (the East- and West-types) in *L*. *nigripes* that seem to have originated from independent polyploidization events. Based on the limited number of recombinants between the East- and West-types and the partial reduction of spore fertility in F_1_ hybrids, it is suggested that the divergence between the East- and West-types represents an incipient stage of speciation with incomplete reproductive isolation. Although the genetic distinctness and isolation between them could suggest that they represent evolutionary distinct lineages respectively, it is concluded that they should be still treated as single species considering the existence of recombinants and morphological indistinctness.

Given that the East- and West-types of *L*. *nigripes* originated allopatrically during the last glacial period, we need to consider that reproductive isolation between the two types developed rapidly, i.e., on the order of tens of thousands of years. Genetic and epigenetic studies of polyploidy have demonstrated that gene silencing and chromosome rearrangement occur immediately following polyploidization [100–103], even within the first few generations in synthesized *Brassica* polyploids [104]. Such gene silencing after polyploidization has also been reported in allopolyploid ferns [105]. Werth and Windham [106] proposed a model in which reciprocal silencing at each of the duplicated loci of the same gene leads to reproductive isolation between allopatric polyploid populations. Future studies on the genetic basis of the intrinsic reproductive isolation between the East- and West-types of *L*. *nigripes* would greatly contribute to the understanding of allopatric speciation at the polyploid level. Intriguingly, heterozygous individuals within each type showed no reduction in the proportion of normal spores, although these individuals could be generated from hybridization between individuals of independent origins within each type. This result suggests that independent origins *per se* did not contribute to the development of reproductive isolation, but rather, allopatric formation and distinct genetic contribution from parental species as a consequence of independent origins could be responsible for speciation between allopolyploid lineages of independent origin. This is consistent with hypotheses previously presented by Soltis et al. [8] and Symonds et al. [23].

## Supporting information

S1 Fig Results of InStruct analysis for trimmed dataset. The proportion of the membership coefficient of 352 individuals in the 51 populations for each of the inferred clusters for K = 2–5 defined using Bayesian clustering in InStruct analysis. Each individual is shown as a column, and populations are separated from each other by a bold black line. Numerals at the bottom indicate population numbers.
(TIF)

S2 Fig Pie graphs showing frequencies of MLGs in each geographic region. Numbers around pie chart indicate MLG numbers, respectively. Color in slice reflects the group of MLGs; blue, East-type; yellow, West-type; green, F_1_ hybrid between East- and West-type; light green, recombinant.
(TIF)

S3 Fig Allelic richness for East- and West-types in each region. Black and grey bars indicate East- and West-type, respectively.
(TIF)

S4 Fig ML phylogenetic trees of plastid region, *rps4-trnS*. Thickest lines indicate strong support (MLBS > = 70, BIPP = 1.0), middle thick lines indicate moderate support (MLBS > = 70, BIPP = 0.99) and thin lines indicate weak support (MLBS < 70 or BIPP < 0.99). Blue and yellow colors indicate East- and West-type individuals, respectively. Individuals connected to each other with grey line are individuals from same population.
(TIF)

S1 Table Locations of the sampled populations of *Lepisorus nigripes*. (DOC)

S2 Table Voucher information for closely related species used in the phylogenetic analysis. (DOC)

S3 Table Accession numbers of nucleotide sequences of *Lepisorus nigripes* and its related species used in this study. (DOC)

S4 Table Information for sample ID, voucher specimen, multilocus genotype, plastid haplotype, and spore fertility of *Lepisorus nigripes*. In type column, each sample was classified into East-type, West-type, F_1_ and Recombinant based on the result in InStruct (see Materials and Methods and Result in the article).
(DOC)

S5 Table Values of ΔK, MedMeaK, MaxMeaK, MedMedK and MaxMedK for Instruct result for full dataset and trimmed dataset. (DOC)
