# Supplementary figures and images for: Independent allopatric polyploidizations shaped the geographical structure and initial stage of reproductive isolation in an allotetraploid fern, Lepisorus nigripes (Polypodiaceae)
Source: PLoS One. 2020 May 20;15(5):e0233095. doi: 10.1371/journal.pone.0233095 (PMC7239481; doi:10.1371/journal.pone.0233095)

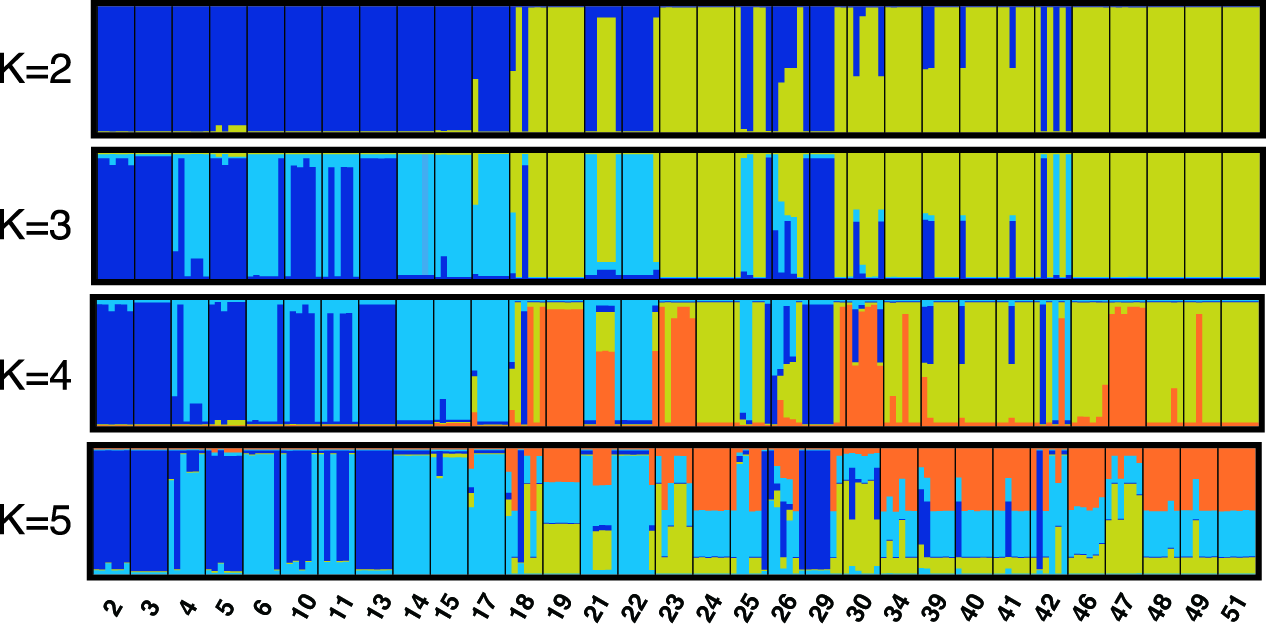

Supplement: S1 Fig — The proportion of the membership coefficient of 352 individuals in the 51 populations for each of the inferred clusters for K = 2–5 defined using Bayesian clustering in InStruct analysis. Each individual is shown as a column, and populations are separated from each other by a bold black line. Numerals at the bottom indicate population numbers. (TIF) [file pone.0233095.s001.tif]

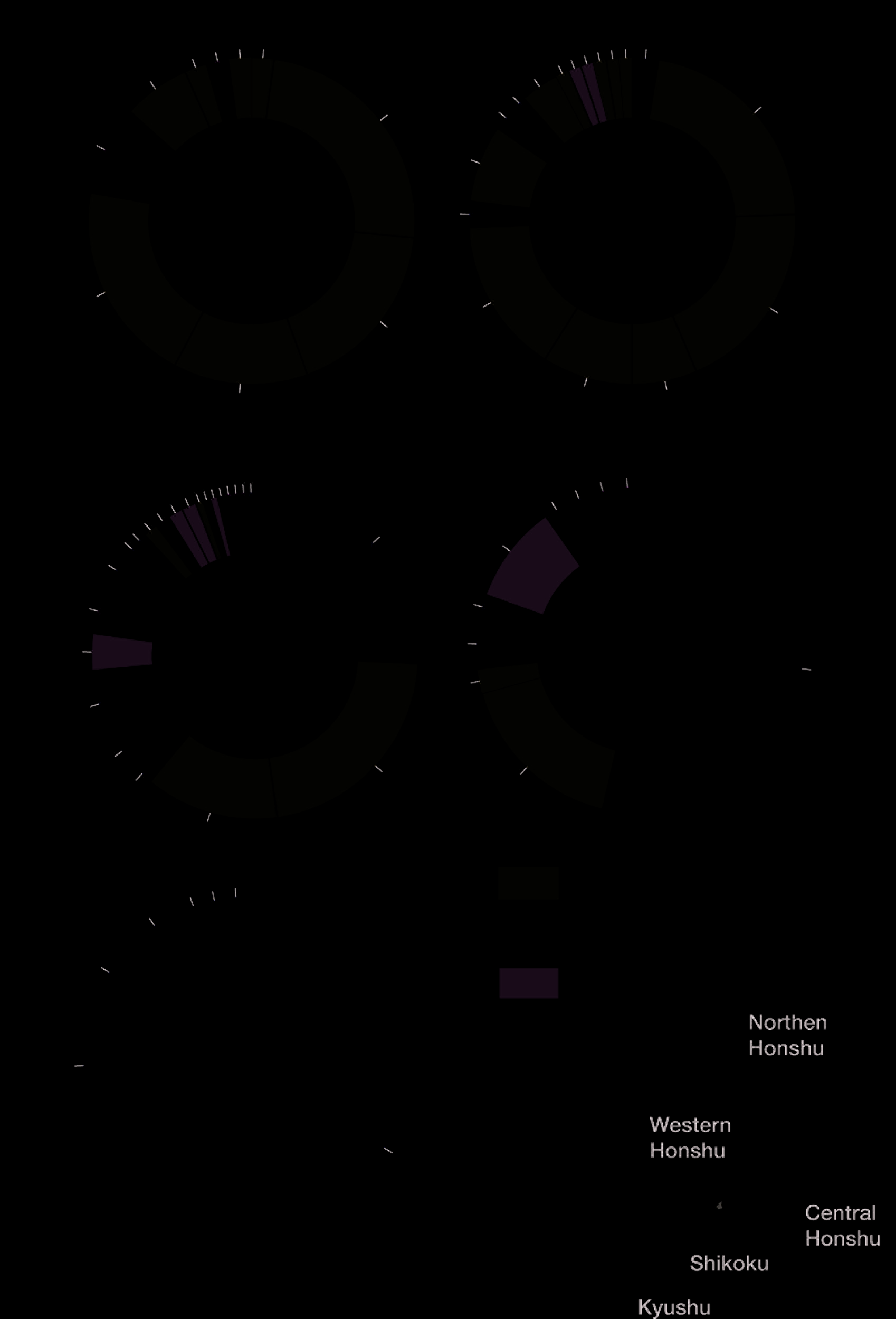

Supplement: S2 Fig — Numbers around pie chart indicate MLG numbers, respectively. Color in slice reflects the group of MLGs; blue, East-type; yellow, West-type; green, F1 hybrid between East- and West-type; light green, recombinant. (TIF) [file pone.0233095.s002.tif]

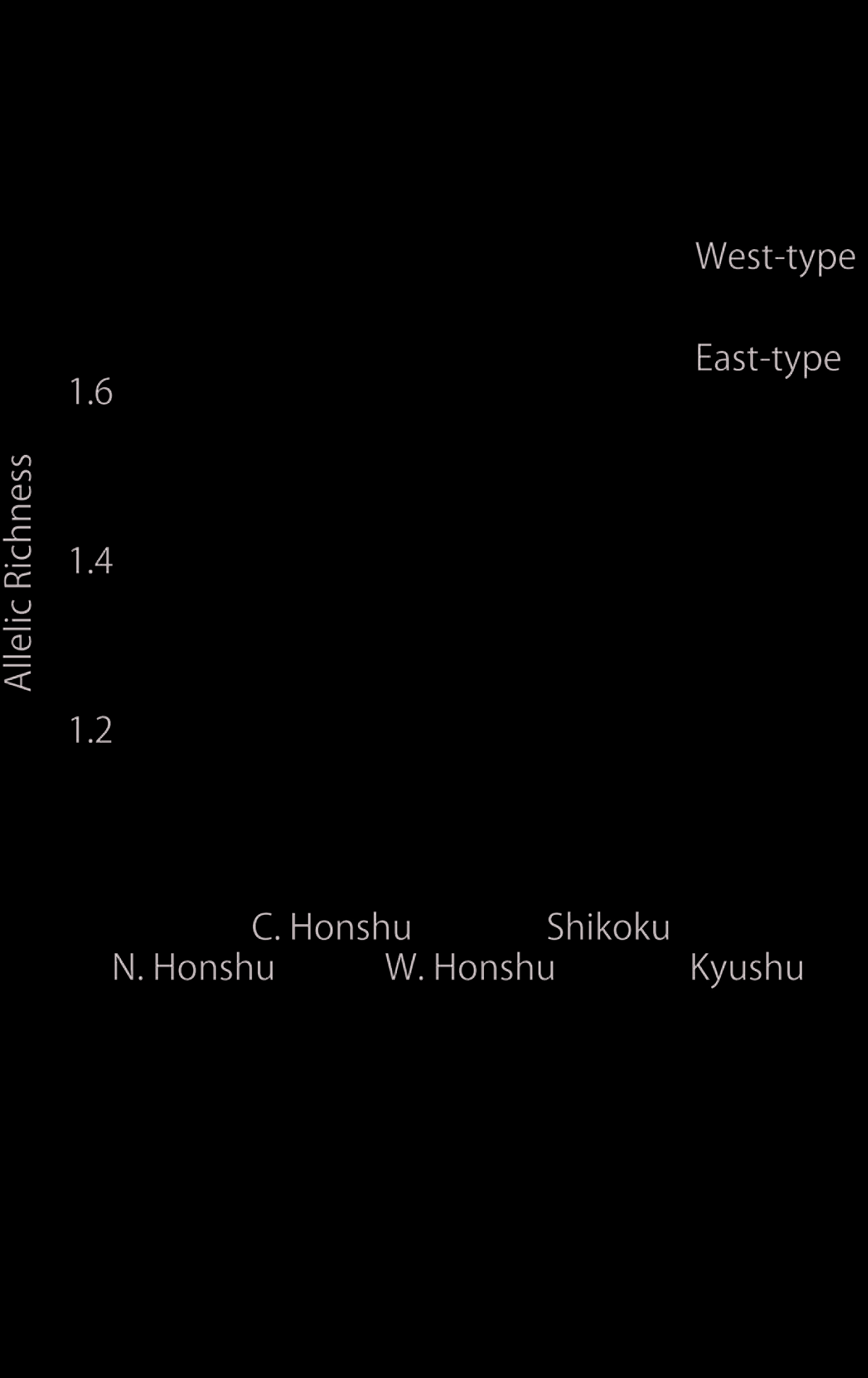

Supplement: S3 Fig — Black and grey bars indicate East- and West-type, respectively. (TIF) [file pone.0233095.s003.tif]

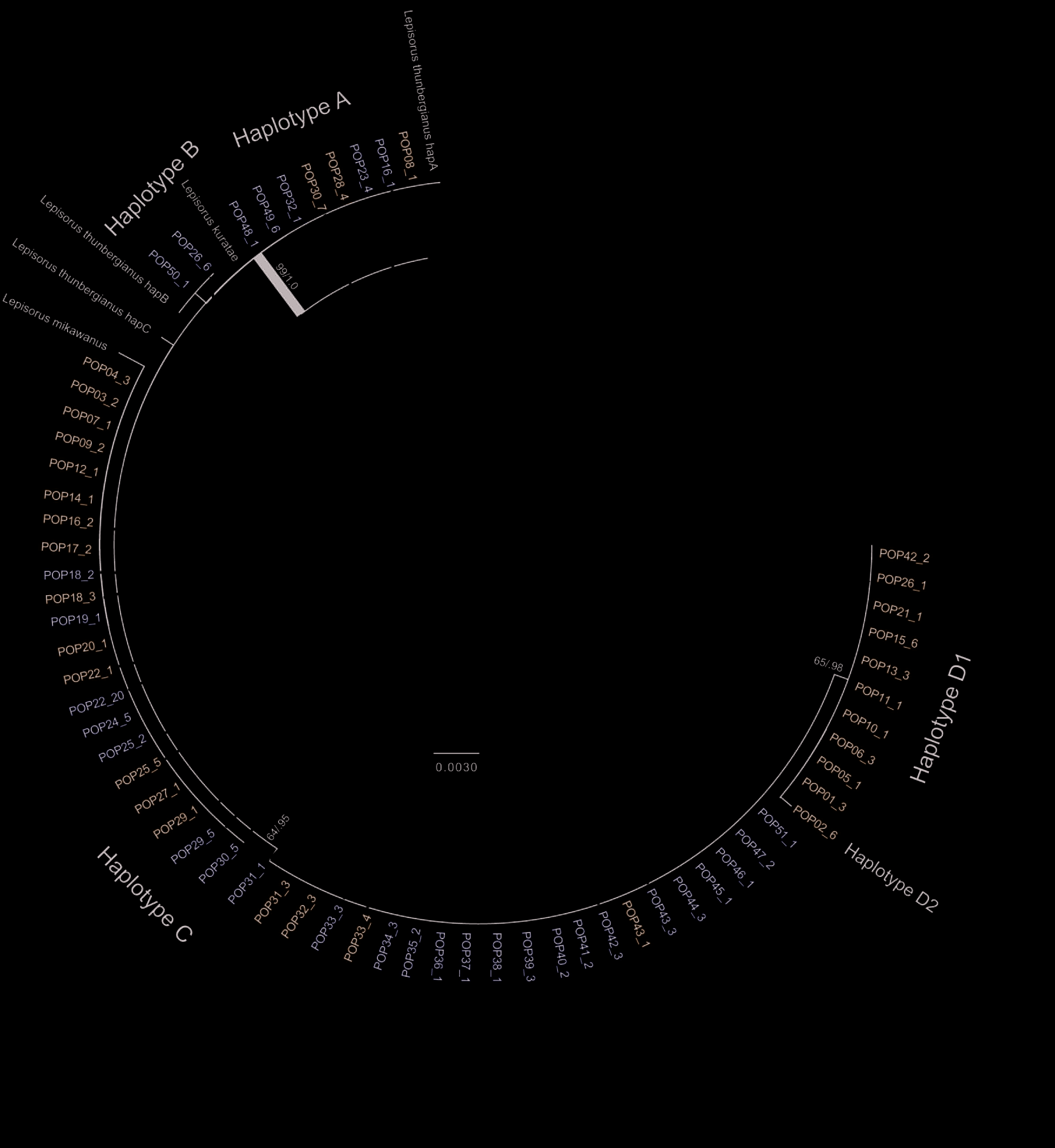

Supplement: S4 Fig — Thickest lines indicate strong support (MLBS > = 70, BIPP = 1.0), middle thick lines indicate moderate support (MLBS > = 70, BIPP = 0.99) and thin lines indicate weak support (MLBS < 70 or BIPP < 0.99). Blue and yellow colors indicate East- and West-type individuals, respectively. Individuals connected to each other with grey line are individuals from same population. (TIF) [file pone.0233095.s004.tif]
