# Supplementary material for: Independent allopatric polyploidizations shaped the geographical structure and initial stage of reproductive isolation in an allotetraploid fern, Lepisorus nigripes (Polypodiaceae)
Source: PLoS One. 2020 May 20;15(5):e0233095. doi: 10.1371/journal.pone.0233095 (PMC7239481; doi:10.1371/journal.pone.0233095)
Supplement: S1 Table — (DOC) [file pone.0233095.s005.doc]

**S1 Table.** Sampling location for each population of *Lepisorus nigripes* used in this study

| **Population code** | **Location** |
| --- | --- |
| 1 | Aomori Pref., Nishitsugaru-shi, Hukaura |
| 2 | Akita Pref., Senhoku-shi, Lake Tazawa |
| 3 | Iwate Pref., Ichinoseki-shi, Genbi |
| 4 | Iwate Pref., Ichinoseki-shi, Yamanometate |
| 5 | Niigata Pref, Nishikambara-gun, Yahiko |
| 6 | Fukushima Pref., Kouriyama-shi, Tamura |
| 7 | Tokyo Pref., Kita-ku, Oji |
| 8 | Chiba Pref., Futtsu-shi, Minato |
| 9 | Kanagawa Pref., Kamakura-shi, Junisho |
| 10 | Kanagawa Pref., Isehara-shi, Mt. Oyama |
| 11 | Tokyo Pref., Nishitama-shi, Okutama |
| 12 | Yamanashi Pref, Hokuto-shi, Sudama |
| 13 | Yamanashi Pref., Hokuto-shi, Nagasaka |
| 14 | Nagano Pref., Matsumoto-shi, Azumi |
| 15 | Gifu Pref., Takayama-shi, Kunifu |
| 16 | Toyama Pref., Takaoka-shi, Nishihirotani |
| 17 | Toyama Pref., Toyama-shi, Mt. Kureha |
| 18 | Ishikawa Pref, Kanazawa-shi, Utastu |
| 19 | Mie Pref., Yokkaichi-shi, Unomori |
| 20 | Mie Pref., Tsu-shi, Igakaido |
| 21 | Mie Pref., Owase-shi, Mikisato |
| 22 | Nara Pref., Yoshino-gun, Kamikitayama |
| 23 | Wakayama Pref., Shingu-shi |
| 24 | Wakayama Pref., Shingu-shi |
| 25 | Wakayama Pref., Ito-gun, Koya |
| 26 | Wakayama Pref., Ito-gun, Katsuragi |
| 27 | Wakayama Pref., Hashimoto-shi, Koyaguti |
| 28 | Wakayama Pref., Hashimoto-shi, Yamada |
| 29 | Osaka Pref., Kawachinagano-shi, Koyakaidou |
| 30 | Osaka Pref., Kawachinagano-shi, Nagaredani |
| 31 | Osaka Pref., Kawachinagano-shi, Iwase |
| 32 | Nara Pref., Gojo-shi, Mt. Katsuragi |
| 33 | Nara Pref., Nara-shi, Kitatsubaocho |
| 34 | Kyoto Pref., Miyazu-shi, Higatani |
| 35 | Hyogo Pref., Kobe-shi, Mt. Rokko |
| 36 | Hyogo Pref., Asago-shi, Santoucho |
| 37 | Tottori Pref., Yazu-gun, Yazucho |
| 38 | Okayama Pref., Takahashi-shi, Bichucho |
| 39 | Hiroshima Pref., Hiroshima-shi, Asacho |
| 40 | Tokushima Pref., Mima-gun, Wakicho |
| 41 | Tokushima Pref., Mima-gun, Tsurugicho |
| 42 | Tokushima Pref., Mima-gun, Tsurugicho |
| 43 | Tokushima Pref., Miyoshi-shi, Higashiiyason |
| 44 | Tokushima Pref., Miyoshi-shi, Nishiiyason |
| 45 | Kochi Pref., Tosa-shi, Tosacho |
| 46 | Oita Pref., Nakatsu-shi, Yabakei |
| 47 | Fukuoka Pref., Buzen-shi, Kubote |
| 48 | Fukuoka Pref., Fukuoka-shi, Iiba |
| 49 | Fukuoka Pref., Itozima-shi, Mt. Ihara |
| 50 | Saga Pref., Fujizu-shi, Mt. Tara |
| 51 | Kumamoto Pref., Aso-shi, Takamori |
