# Supplementary material for: Independent allopatric polyploidizations shaped the geographical structure and initial stage of reproductive isolation in an allotetraploid fern, Lepisorus nigripes (Polypodiaceae)
Source: PLoS One. 2020 May 20;15(5):e0233095. doi: 10.1371/journal.pone.0233095 (PMC7239481; doi:10.1371/journal.pone.0233095)
Supplement: S2 Table — (DOC) [file pone.0233095.s006.doc]

**S2 Table**. Voucher information for closely related species used in Phylogenetic analysis

| Species name | Voucher number | Location |
| --- | --- | --- |
| *Lepisorus thunbergianus* | T. Fujiwara 150221-9 | Kanagawa Pref., Kamakura-shi, Junisho |
|  | T. Fujiwara 150227-6 | Chiba Pref., Futtsu-shi, Minato |
|  | T. Fujiwara 150227-7 | Chiba Pref., Futtsu-shi, Minato |
|  | T. Fujiwara 150317-1 | Saga Pref., Fujitsu-shi, Mt. Tara |
|  | T. Fujiwara 150317-2 | Kumamoto Pref., Kikuchi-shi, Ichihara |
|  | T. Fujiwara 150317-4 | Kumamoto Pref., Kikuchi-shi, Ichihara |
|  | T. Fujiwara 150317-4 | Kumamoto Pref., Kikuchi-shi, Ichihara |
|  | T. Fujiwara 150322-8 | Kagoshima Pref., Satsuma-shi, Satsuma |
|  | T. Fujiwara 150527-4 | Okayama Pref., Bizen-shi |
|  | T. Fujiwara 151225-14 | Shizuoka Pref., Kamo-gun, Kawazu |
|  | T. Fujiwara 160316-1 | Chiba Pref., Chiba-shi, Inage-ku, Yayoi |
|  | T. Fujiwara 160329-1 | Oita Pref., Bungotakada-shi, Fukkiji |
| *Lepisorus angustus* | T. Fujiwara 160326-1 | Fukushima Pref, Yama-gun, Kitashiobara |
|  | T. Fujiwara 151115-10 | Yamanashi Pref., Hokuto-shi, Sudama |
|  | T. Fujiwara 151115-11 | Yamanashi Pref., Hokuto-shi, Sudama |
|  | T. Fujiwara 151115-2 | Yamanashi Pref., Hokuto-shi, Sudama |
|  | T. Fujiwara 170527-1 | Nara Pref., Yoshino-gun, Kamikitayama, Mt. daihugen |
|  | T. Fujiwara 170527-2 | Nara Pref., Yoshino-gun, Kamikitayama, Mt. daihugen |
|  | T. Fujiwara 170527-3 | Nara Pref., Yoshino-gun, Kamikitayama, Mt. daihugen |
|  | T. Fujiwara 170527-4 | Nara Pref., Yoshino-gun, Kamikitayama, Mt. daihugen |
|  | T. Fujiwara 170527-5 | Nara Pref., Yoshino-gun, Kamikitayama, Mt. daihugen |
|  | T. Fujiwara 170527-6 | Nara Pref., Yoshino-gun, Kamikitayama, Mt. daihugen |
|  | T. Fujiwara 170527-8 | Nara Pref., Yoshino-gun, Kamikitayama, Mt. daihugen |
| *L. tosaensis* | T. Fujiwara 150326-14 | Oita Pref., Nakatsu-shi, Yabakei |
| *L. hachijoensis* | T. Fujiwara 140904-4 | Tokyo Pref., Hachijo Is. |
| *L. onoei* | T. Fujiwara 150325-27 | Fukuoka Pref., Itojima-shi, Mt Ihara |
| *L. kuratae* | T. Fujiwara 150221-5 | Kanagawa Pref., Kamakura-shi, Juunisho |
| *L. uchiyamae* | T. Fujiwara 170703-20 | Kochi Pref., Aki-shi, Shimoyama, Oyamamisaki |
