# Supplementary material for: Independent allopatric polyploidizations shaped the geographical structure and initial stage of reproductive isolation in an allotetraploid fern, Lepisorus nigripes (Polypodiaceae)
Source: PLoS One. 2020 May 20;15(5):e0233095. doi: 10.1371/journal.pone.0233095 (PMC7239481; doi:10.1371/journal.pone.0233095)
Supplement: S3 Table — (DOC) [file pone.0233095.s007.doc]

**S3 Table.** Accession numbers of sequences used in this study

|  | ***PgiC*** | | | | ***GapCp*** | | | | ***pTpi*** | | | | ***Rps4-trnS*** | |
| --- | --- | --- | --- | --- | --- | --- | --- | --- | --- | --- | --- | --- | --- | --- |
|  | ***PgiC-thu*** | | ***PgiC-ang*** | | ***GapCp-thu*** | | ***GapCp -ang*** | | ***pTpi-thu*** | | ***pTpi-ang*** | |
| ***Lepisorus nigripes*** | Thu-1 | LC360559 | Ang-1 | LC360553 | Thu-1 | LC360528 | Ang-1 | LC360536 | Thu-1 | LC360525 | Ang-1 | LC360515 | Hap_A | LC367225 |
|  | Thu-2 | LC360564 | Ang-2 | LC360554 | Thu-2 | LC360529 | Ang-2 | LC360535 | Thu-2 | LC360516 | Ang-2 | LC360524 | Hap_B | LC367226 |
|  | Thu-3 | LC360572 |  |  | Thu-3 | LC360542 | Ang-3 | LC360537 |  |  |  |  | Hap_C | LC367227 |
|  | Thu-4 | LC360580 |  |  | Thu-4 | LC360530 |  |  |  |  |  |  | Hap_D1 | LC367228 |
|  | Thu-5 | LC360560 |  |  |  |  |  |  |  |  |  |  | Hap_D2 | LC367229 |
|  | Thu-6 | LC360575 |  |  |  |  |  |  |  |  |  |  |  |  |
|  | Thu-7 | LC360568 |  |  |  |  |  |  |  |  |  |  |  |  |
|  | Thu-8 | LC360576 |  |  |  |  |  |  |  |  |  |  |  |  |
|  | Thu-9 | LC360570 |  |  |  |  |  |  |  |  |  |  |  |  |
|  | Thu-10 | LC360574 |  |  |  |  |  |  |  |  |  |  |  |  |
|  | Thu-11 | LC360563 |  |  |  |  |  |  |  |  |  |  |  |  |
|  | Thu-12 | LC360562 |  |  |  |  |  |  |  |  |  |  |  |  |
| ***L. thunbergianus*** | Allele 1 | | LC360571 | | Allele 1 | | LC360531 | | Allele 1 | | LC360518 | | Hap_A | LC332090 |
|  | Allele 2 | | LC360567 | | Allele 2 | | LC360532 | | Allele 2 | | LC360519 | | Hap_B | XXXXXX |
|  | Allele 3 | | LC360584 | | Allele 3 | | LC360547 | | Allele 3 | | LC360520 | | Hap_C | LC332093 |
|  | Allele 4 | | LC360569 | | Allele 4 | | LC360533 | | Allele 4 | | LC360526 | |  |  |
|  | Allele 5 | | LC360577 | | Allele 5 | | LC360543 | | Allele 5 | | LC360521 | |  |  |
|  | Allele 6 | | LC360578 | | Allele 6 | | LC360544 | |  | |  | |  |  |
|  | Allele 7 | | LC360583 | | Allele 7 | | LC360538 | |  | |  | |  |  |
|  | Allele 8 | | LC360585 | | Allele 8 | | LC360534 | |  | |  | |  |  |
| ***L. thunbergianus*** | Allele 9 | | LC360579 | | Allele 9 | | LC373183 | |  | |  | |  | |
|  | Allele 10 | | LC360582 | | Allele 10 | | LC373184 | |  | |  | |  | |
|  | Allele 11 | | LC360565 | | Allele 11 | | LC373185 | |  | |  | |  | |
|  |  | |  | | Allele 12 | | LC373186 | |  | |  | |  | |
|  |  | |  | | Allele 13 | | LC373187 | |  | |  | |  | |
|  |  | |  | | Allele 14 | | LC373188 | |  | |  | |  | |
|  |  | |  | | Allele 15 | | LC373189 | |  | |  | |  | |
|  |  | |  | | Allele 16 | | LC373190 | |  | |  | |  | |
| ***L. angustus* (Japan)** | Allele 1 | | LC360551 | | Allele 1 | | LC360539 | | Allele 1 | | LC360508 | | LC332087 | |
|  | Allele 2 | | LC360552 | | Allele 2 | | LC360545 | | Allele 2 | | LC360509 | |
|  | Allele 3 | | LC360573 | | Allele 3 | | LC360548 | | Allele 3 | | LC360510 | |
|  | Allele 4 | | LC360556 | | Allele 4 | | LC360540 | | Allele 4 | | LC360511 | |
|  | Allele 5 | | LC360555 | | Allele 5 | | LC360541 | | Allele 5 | | LC360523 | |
|  | Allele 6 | | LC360557 | |  | |  | |  | |  | |
| ***L. affinia*** |  | | - | |  | | - | |  | | - | | GQ256328 | |
| ***L. angustus* (China)** |  | | - | |  | | - | |  | | - | | GQ256364 | |
| ***L. boninensis*** |  | | - | |  | | - | |  | | - | | GQ256335 | |
| ***L. confluens*** |  | | - | |  | | - | |  | | - | | GQ256337 | |
| ***L. contortus*** |  | | - | |  | | - | |  | | - | | GQ256338 | |
| ***L. elegans*** |  | | - | |  | | - | |  | | - | | GQ256342 | |
| ***L. hachijoensis*** |  | | LC360558 | |  | | LC373158 | |  | | LC360512 | | LC332084 | |
| ***L. lewissi*** |  | | - | |  | | - | |  | | - | | GQ256347 | |
| ***L. lineariformis*** |  | | - | |  | | - | |  | | - | | GQ256350 | |
| ***L. kuratae*** | A | | LC360586 | | A | | LC373178 | | A | | LC360513 | | LC332106 | |
|  | B | | LC360581 | | B | | LC373179 | | B | | LC360514 | |
| ***L. medogensis*** |  | | - | |  | | - | |  | | - | | GQ256356 | |
| ***L. mikawanus*** |  | | - | |  | | - | |  | | - | | LC332112 | |
| ***L. monilisorus*** |  | | - | |  | | - | |  | | - | | GQ256357 | |
| ***L. obscurevenulosus*** |  | | - | |  | | - | |  | | - | | GQ256360 | |
| ***L. oligolepidus*** |  | | - | |  | | - | |  | | - | | GQ256361 | |
| ***L. onoei*** |  | | LC360561 | |  | | LC360550 | |  | | LC360517 | | LC332083 | |
| ***L. sinensis*** |  | | - | |  | | - | |  | | - | | GQ256370 | |
| ***L. sordidus*** |  | | - | |  | | - | |  | | - | | GQ256371 | |
| ***L. subconfluens*** |  | | - | |  | | - | |  | | - | | GQ256373 | |
| ***L. suboligolepidus*** |  | | - | |  | | - | |  | | - | | GQ256376 | |
| ***L. tibeticus*** |  | | - | |  | | - | |  | | - | | GQ256383 | |
| ***L. tosaensis*** | A | | LC360566 | |  | | LC360546 | |  | | - | | LC332085 | |
|  | B | | LC360587 | |  | |  | |  | |  | |
| ***L. uchiyamae*** |  | | LC373182 | |  | | LC373181 | |  | | LC360527 | | GQ256386 | |
| ***L. ussuriensis*** |  | | - | |  | | - | |  | | - | | GQ256388 | |
