# Supplementary material for: Independent allopatric polyploidizations shaped the geographical structure and initial stage of reproductive isolation in an allotetraploid fern, Lepisorus nigripes (Polypodiaceae)
Source: PLoS One. 2020 May 20;15(5):e0233095. doi: 10.1371/journal.pone.0233095 (PMC7239481; doi:10.1371/journal.pone.0233095)
Supplement: S4 Table — In type column, each sample was classified into East-type, West-type, F1 and Recombinant based on the result in InStruct (see Materials and Methods and Result in the article). (DOC) [file pone.0233095.s008.doc]

**S4 Table.** Information for sample ID, voucher specimen, multilocus genotype, plastid haplotype, and spore fertility of *Lepisorus nigripes.* In type column, each sample was classified into East, West, F_1_ and Recombinant based on the result in InStruct (see Materials and Methods and Result in the article).

| Location code | sample ID | Voucher specimen | Spore fertility | cpDNA haplotype | MLG | Type |
| --- | --- | --- | --- | --- | --- | --- |
| 1 | 1-1 | T. Fujiwara 160605-1 |  |  | 5 | East |
| 1 | 1-2 | T. Fujiwara 160605-2 |  |  | 3 | East |
| 1 | 1-3 | T. Fujiwara 160605-3 |  | D1 | 27 | East |
| 1 | 1-4 | T. Fujiwara 160605-4 |  |  | 48 | East |
| 2 | 2-1 | T. Fujiwara 160607-1 |  |  | 3 | East |
| 2 | 2-2 | T. Fujiwara 160607-2 |  |  | 3 | East |
| 2 | 2-3 | T. Fujiwara 160607-3 |  |  | 3 | East |
| 2 | 2-4 | T. Fujiwara 160607-4 |  |  | 5 | East |
| 2 | 2-5 | T. Fujiwara 160607-5 | 0.994 |  | 3 | East |
| 2 | 2-6 | T. Fujiwara 160607-6 |  | D2 | 3 | East |
| 2 | 2-7 | T. Fujiwara 160607-7 |  |  | 3 | East |
| 2 | 2-8 | T. Fujiwara 160607-8 | 0.773 |  | 37* | East* |
| 2 | 2-9 | T. Fujiwara 160607-9 |  |  | 3 | East |
| 2 | 2-10 | T. Fujiwara 160607-10 |  |  | 5 | East |
| 2 | 2-11 | T. Fujiwara 160607-11 |  |  | 5 | East |
| 3 | 3-1 | T. Fujiwara 160608-10 |  |  | 11 | East |
| 3 | 3-2 | T. Fujiwara 160608-11 |  | C | 11 | East |
| 3 | 3-3 | T. Fujiwara 160608-12 |  |  | 11 | East |
| 3 | 3-4 | T. Fujiwara 160608-13 |  |  | 11 | East |
| 3 | 3-5 | T. Fujiwara 160608-14 |  |  | 11 | East |
| 3 | 3-6 | T. Fujiwara 160608-15 |  |  | 11 | East |
| 3 | 3-7 | T. Fujiwara 160608-16 |  |  | 11 | East |
| 3 | 3-8 | T. Fujiwara 160608-17 |  |  | 40 | Recombinant |
| 3 | 3-9 | T. Fujiwara 160608-18 |  |  | 11 | East |
| 3 | 3-10 | T. Fujiwara 160608-19 |  |  | 11 | East |
| 4 | 4-1 | T. Fujiwara 160608-1 | 0.950 | 0 | 4 | East |
| 4 | 4-2 | T. Fujiwara 160608-2 |  |  | 16* | East |
| 4 | 4-3 | T. Fujiwara 160608-3 |  | C | 4 | East |
| 4 | 4-4 | T. Fujiwara 160608-4 |  |  | 3 | East |
| 4 | 4-5 | T. Fujiwara 160608-5 |  |  | 4 | East |
| 4 | 4-6 | T. Fujiwara 160608-6 | 0.757 |  | 16* | East |
| 4 | 4-7 | T. Fujiwara 160608-7 | 0.805 |  | 16* | East |
| 4 | 4-8 | T. Fujiwara 160608-8 |  |  | 4 | East |
| 5 | 5-1 | T. Fujiwara 150908-1 |  | D1 | 5 | East |
| 5 | 5-2 | T. Fujiwara 150908-2 |  |  | 3 | East |
| 5 | 5-3 | T. Fujiwara 150908-3 |  |  | 15 | Recombinant |
| 5 | 5-4 | T. Fujiwara 150908-4 |  |  | 5 | East |
| 5 | 5-5 | T. Fujiwara 150908-5 |  |  | 15 | Recombinant |
| 5 | 5-6 | T. Fujiwara 150908-6 |  |  | 15 | Recombinant |
| 5 | 5-7 | T. Fujiwara 150908-7 |  |  | 15 | Recombinant |
| 6 | 6-1 | T. Fujiwara 150706-3 |  |  | 4 | East |
| 6 | 6-2 | T. Fujiwara 150706-4 |  |  | 2 | East |
| 6 | 6-3 | T. Fujiwara 161029-1 |  | D1 | 4 | East |
| 6 | 6-4 | T. Fujiwara 161029-2 | 0.930 |  | 4 | East |
| 6 | 6-5 | T. Fujiwara 161029-3 |  |  | 4 | East |
| 6 | 6-6 | T. Fujiwara 161029-5 |  |  | 3 | East |
| 7 | 7-1 | T. Fujiwara 150829-1 |  | C | 3 | East |
| 7 | 7-2 | T. Fujiwara 150829-3 |  |  | 3 | East |
| 7 | 7-3 | T. Fujiwara 150829-4 |  |  | 3 | East |
| 8 | 8-1 | T. Fujiwara 150227-1 |  | A | 2 | East |
| 8 | 8-2 | T. Fujiwara 150227-2 |  |  | 2 | East |
| 8 | 8-3 | T. Fujiwara 150227-3 |  |  | 2 | East |
| 8 | 8-4 | T. Fujiwara 150227-4 |  |  | 2 | East |
| 8 | 8-5 | T. Fujiwara 150227-5 |  |  | 2 | East |
| 9 | 9-1 | T. Fujiwara 150221-3 |  |  | 6 | East |
| 9 | 9-2 | T. Fujiwara 150221-4 |  | C | 6 | East |
| 9 | 9-3 | T. Fujiwara 150221-12 |  |  | 6 | East |
| 9 | 9-4 | T. Fujiwara 150221-13 |  |  | 6 | East |
| 9 | 9-5 | T. Fujiwara 150221-14 |  |  | 6 | East |
| 9 | 9-6 | T. Fujiwara 150221-18 |  |  | 2 | East |
| 10 | 10-1 | T. Fujiwara 150517-18 |  | D1 | 4 | East |
| 10 | 10-2 | T. Fujiwara 150517-19 | 1.000 |  | 35* | East |
| 10 | 10-3 | T. Fujiwara 150517-20 |  |  | 3 | East |
| 10 | 10-4 | T. Fujiwara 150517-21 | 0.742 |  | 6 | East |
| 10 | 10-5 | T. Fujiwara 150517-23 |  |  | 6 | East |
| 10 | 10-6 | T. Fujiwara 150517-26 |  |  | 3 | East |
| 10 | 10-7 | T. Fujiwara 150517-27 |  |  | 4 | East |
| 10 | 10-8 | T. Fujiwara 150517-28 |  |  | 6 | East |
| 10 | 10-9 | T. Fujiwara 150517-29 |  |  | 19 | East |
| 10 | 10-10 | T. Fujiwara 150517-30 |  |  | 26* | East |
| 10 | 10-11 | T. Fujiwara 150517-31 |  |  | 3 | East |
| 11 | 11-1 | T. Fujiwara 170503-1 |  | D1 | 4 | East |
| 11 | 11-2 | T. Fujiwara 170503-2 |  |  | 19 | East |
| 11 | 11-3 | T. Fujiwara 170503-3 |  |  | 6 | East |
| 11 | 11-4 | T. Fujiwara 170503-5 |  |  | 4 | East |
| 11 | 11-5 | T. Fujiwara 170503-7 |  |  | 3 | East |
| 11 | 11-6 | T. Fujiwara 170503-8 |  |  | 45 | East |
| 11 | 11-7 | T. Fujiwara 170503-9 |  |  | 4 | East |
| 11 | 11-8 | T. Fujiwara 170503-10 |  |  | 6 | East |
| 11 | 11-9 | T. Fujiwara 170503-11 |  |  | 6 | East |
| 11 | 11-10 | T. Fujiwara 170503-12 |  |  | 19 | East |
| 12 | 12-1 | T. Fujiwara 151115-1 |  | C | 6 | East |
| 12 | 12-2 | T. Fujiwara 151115-2 |  |  | 2 | East |
| 12 | 12-3 | T. Fujiwara 151115-3 |  |  | 2 | East |
| 12 | 12-4 | T. Fujiwara 151115-4 |  |  | 2 | East |
| 13 | 13-1 | T. Fujiwara 160406-1 | 0.907 |  | 3 | East |
| 13 | 13-2 | T. Fujiwara 160406-2 |  |  | 3 | East |
| 13 | 13-3 | T. Fujiwara 160406-3 |  | D1 | 3 | East |
| 13 | 13-4 | T. Fujiwara 160406-4 |  |  | 3 | East |
| 13 | 13-5 | T. Fujiwara 160406-5 |  |  | 3 | East |
| 13 | 13-6 | T. Fujiwara 160406-6 |  |  | 3 | East |
| 13 | 13-7 | T. Fujiwara 160406-7 |  |  | 3 | East |
| 13 | 13-8 | T. Fujiwara 160406-9 |  |  | 3 | East |
| 14 | 14-1 | T. Fujiwara 160807-1 |  | C | 2 | East |
| 14 | 14-2 | T. Fujiwara 160807-2 |  |  | 2 | East |
| 14 | 14-3 | T. Fujiwara 160807-3 |  |  | 2 | East |
| 14 | 14-4 | T. Fujiwara 160807-4 |  |  | 2 | East |
| 14 | 14-5 | T. Fujiwara 160807-5 |  |  | 2 | East |
| 14 | 14-6 | T. Fujiwara 160807-6 |  |  | 2 | East |
| 14 | 14-7 | T. Fujiwara 160807-7 |  |  | 2 | East |
| 15 | 15-1 | T. Fujiwara 160308-1 | 1.000 |  | 13 | East |
| 15 | 15-2 | T. Fujiwara 160308-2 | 0.969 |  | 34* | East |
| 15 | 15-3 | T. Fujiwara 160308-3 |  |  | 13 | East |
| 15 | 15-4 | T. Fujiwara 160308-4 |  |  | 13 | East |
| 15 | 15-5 | T. Fujiwara 160308-5 |  |  | 13 | East |
| 15 | 15-6 | T. Fujiwara 160308-6 |  | D1 | 13 | East |
| 15 | 15-7 | T. Fujiwara 160308-8 |  |  | 13 | East |
| 16 | 16-1 | T. Fujiwara 150915-1 |  | A | 17 | West |
| 16 | 16-2 | T. Fujiwara 150915-2 |  | C | 2 | East |
| 16 | 16-3 | T. Fujiwara 150915-3 |  |  | 17 | West |
| 17 | 17-1 | T. Fujiwara 170821-9 | 0.457 |  | 32* | F1 |
| 17 | 17-2 | T. Fujiwara 170821-10 | 0.938 | C | 5 | East |
| 17 | 17-3 | T. Fujiwara 170821-11 |  |  | 5 | East |
| 17 | 17-4 | T. Fujiwara 170821-12 |  |  | 5 | East |
| 17 | 17-5 | T. Fujiwara 170821-13 |  |  | 5 | East |
| 17 | 17-6 | T. Fujiwara 170821-14 |  |  | 5 | East |
| 17 | 17-7 | T. Fujiwara 170821-15 |  |  | 5 | East |
| 18 | 18-1 | T. Fujiwara 170821-1 | 0.475 |  | 33* | F1 |
| 18 | 18-2 | T. Fujiwara 170821-2 |  | C | 1 | West |
| 18 | 18-3 | T. Fujiwara 170821-3 | 0.859 | C | 5 | East |
| 18 | 18-4 | T. Fujiwara 170821-4 |  |  | 8 | West |
| 18 | 18-5 | T. Fujiwara 170821-5 |  |  | 18 | West |
| 18 | 18-6 | T. Fujiwara 170821-6 |  |  | 1 | West |
| 18 | 18-7 | T. Fujiwara 170821-7 |  |  | 8 | West |
| 19 | 19-1 | T. Fujiwara 161203-8 |  | C | 14 | West |
| 19 | 19-2 | T. Fujiwara 161203-9 |  |  | 14 | West |
| 19 | 19-3 | T. Fujiwara 161203-10 |  |  | 14 | West |
| 19 | 19-4 | T. Fujiwara 161203-11 |  |  | 14 | West |
| 19 | 19-5 | T. Fujiwara 161203-12 |  |  | 14 | West |
| 19 | 19-6 | T. Fujiwara 161203-13 |  |  | 14 | West |
| 20 | 20-1 | T. Fujiwara 161203-1 |  | C | 2 | East |
| 20 | 20-2 | T. Fujiwara 161203-2 |  |  | 30 | East |
| 20 | 20-3 | T. Fujiwara 161203-4 |  |  | 2 | East |
| 21 | 21-1 | T. Fujiwara 161201-1 |  | D1 | 2 | East |
| 21 | 21-2 | T. Fujiwara 161201-2 |  |  | 2 | East |
| 21 | 21-3 | T. Fujiwara 161201-4 |  |  | 12 | Recombinant |
| 21 | 21-4 | T. Fujiwara 161201-5 |  |  | 12 | Recombinant |
| 21 | 21-5 | T. Fujiwara 161201-6 |  |  | 12 | Recombinant |
| 21 | 21-6 | T. Fujiwara 161201-7 |  |  | 12 | Recombinant |
| 21 | 21-7 | T. Fujiwara 161201-8 |  |  | 12 | Recombinant |
| 21 | 21-8 | T. Fujiwara 161201-9 |  |  | 2 | East |
| 22 | 22-1 | T. Fujiwara 170526-2 |  | C | 2 | East |
| 22 | 22-2 | T. Fujiwara 170526-4 |  |  | 2 | East |
| 22 | 22-3 | T. Fujiwara 170526-5 |  |  | 2 | East |
| 22 | 22-4 | T. Fujiwara 170526-6 |  |  | 2 | East |
| 22 | 22-5 | T. Fujiwara 170526-7 |  |  | 2 | East |
| 22 | 22-6 | T. Fujiwara 170526-8 |  |  | 2 | East |
| 22 | 22-7 | T. Fujiwara 170526-9 |  |  | 2 | East |
| 22 | 22-8 | T. Fujiwara 170526-10 |  |  | 2 | East |
| 22 | 22-9 | T. Fujiwara 170526-11 |  |  | 2 | East |
| 22 | 22-10 | T. Fujiwara 170526-12 |  |  | 22* | Recombinant |
| 22 | 22-11 | T. Fujiwara 170526-13 |  |  | 2 | East |
| 22 | 22-12 | T. Fujiwara 170526-14 |  |  | 2 | East |
| 22 | 22-13 | T. Fujiwara 170526-15 |  |  | 2 | East |
| 22 | 22-14 | T. Fujiwara 170526-16 |  |  | 2 | East |
| 22 | 22-15 | T. Fujiwara 170526-17 |  |  | 2 | East |
| 22 | 22-16 | T. Fujiwara 170526-18 |  |  | 2 | East |
| 22 | 22-17 | T. Fujiwara 170526-19 | 1 |  | 2 | East |
| 22 | 22-18 | T. Fujiwara 170526-20 |  |  | 2 | East |
| 22 | 22-19 | T. Fujiwara 170526-21 |  |  | 12 | Recombinant |
| 22 | 22-20 | T. Fujiwara 170526-22 |  | C | 1 | West |
| 22 | 22-21 | T. Fujiwara 170526-24 |  |  | 1 | West |
| 22 | 22-22 | T. Fujiwara 170526-25 | 0.511 |  | 23* | F1 |
| 22 | 22-23 | T. Fujiwara 170526-27 |  |  | 3 | East |
| 22 | 22-24 | T. Fujiwara 170526-28 |  |  | 2 | East |
| 22 | 22-25 | T. Fujiwara 170526-29 |  |  | 2 | East |
| 22 | 22-26 | T. Fujiwara 170526-30 |  |  | 9 | West |
| 22 | 22-27 | T. Fujiwara 170526-31 |  |  | 1 | West |
| 22 | 22-28 | T. Fujiwara 170526-32 |  |  | 2 | East |
| 23 | 23-1 | T. Fujiwara 161202-4 |  |  | 8 | West |
| 23 | 23-2 | T. Fujiwara 161202-5 |  |  | 1 | West |
| 23 | 23-3 | T. Fujiwara 161202-6 |  |  | 9 | West |
| 23 | 23-4 | T. Fujiwara 161202-7 |  |  | 8 | West |
| 23 | 23-5 | T. Fujiwara 161202-8 |  |  | 8 | West |
| 23 | 23-6 | T. Fujiwara 161202-9 |  |  | 9 | West |
| 23 | 23-7 | T. Fujiwara 161202-11 |  |  | 1 | West |
| 24 | 24-1 | T. Fujiwara 161202-13 |  |  | 9 | West |
| 24 | 24-2 | T. Fujiwara 161202-15 |  |  | 1 | West |
| 24 | 24-3 | T. Fujiwara 161202-16 |  |  | 1 | West |
| 24 | 24-4 | T. Fujiwara 161202-19 |  |  | 1 | West |
| 24 | 24-5 | T. Fujiwara 161202-21 |  | C | 1 | West |
| 24 | 24-6 | T. Fujiwara 161202-22 |  |  | 1 | West |
| 24 | 24-7 | T. Fujiwara 161202-23 |  |  | 9 | West |
| 24 | 24-8 | T. Fujiwara 161202-24 | 0.922 |  | 1 | West |
| 25 | 25-1 | T. Fujiwara 160410-1 |  |  | 2 | East |
| 25 | 25-2 | T. Fujiwara 160410-4 |  | C | 1 | West |
| 25 | 25-3 | T. Fujiwara 160410-5 |  |  | 31* | Recombinant |
| 25 | 25-4 | T. Fujiwara 160410-7 |  |  | 2 | East |
| 25 | 25-5 | T. Fujiwara 160410-8 |  | C | 2 | East |
| 25 | 25-6 | T. Fujiwara 160410-9 |  |  | 1 | West |
| 25 | 25-7 | T. Fujiwara 160410-10 |  |  | 1 | West |
| 25 | 25-8 | T. Fujiwara 160410-11 |  |  | 29? | East |
| 26 | 26-1 | T. Fujiwara 170114-30 | 0.843 | D1 | 21* | East |
| 26 | 26-2 | T. Fujiwara 170114-32 |  |  | 22* | Recombinant |
| 26 | 26-3 | T. Fujiwara 170114-33 | 0.899 |  | 21* | East |
| 26 | 26-4 | T. Fujiwara 170114-34 | 0.495 |  | 23* | F1 |
| 26 | 26-5 | T. Fujiwara 170114-35 | 0.440 |  | 10* | F1 |
| 26 | 26-6 | T. Fujiwara 170114-36 | 0.984 | B | 1 | West |
| 26 | 26-7 | T. Fujiwara 170114-37 |  |  | 3 | East |
| 27 | 27-1 | T. Fujiwara 170114-9 |  | C | 3 | East |
| 27 | 27-2 | T. Fujiwara 170114-11 |  |  | 3 | East |
| 27 | 27-3 | T. Fujiwara 170114-13 |  |  | 3 | East |
| 27 | 27-4 | T. Fujiwara 170114-15 |  |  | 2 | East |
| 28 | 28-1 | T. Fujiwara 170114-38 |  |  | 3 | East |
| 28 | 28-2 | T. Fujiwara 170114-39 |  |  | 3 | East |
| 28 | 28-3 | T. Fujiwara 170114-40 |  |  | 3 | East |
| 28 | 28-4 | T. Fujiwara 170114-41 |  | A | 3 | East |
| 28 | 28-5 | T. Fujiwara 170114-42 |  |  | 3 | East |
| 29 | 29-1 | T. Fujiwara 170114-20 |  | C | 3 | East |
| 29 | 29-2 | T. Fujiwara 170114-22 |  |  | 3 | East |
| 29 | 29-3 | T. Fujiwara 170114-23 |  |  | 3 | East |
| 29 | 29-4 | T. Fujiwara 170114-26 |  |  | 3 | East |
| 29 | 29-5 | T. Fujiwara 170114-27 |  | C | 1 | West |
| 29 | 29-6 | T. Fujiwara 170114-28 |  |  | 8 | West |
| 30 | 30-1 | T. Fujiwara 160409-13 |  |  | 18 | West |
| 30 | 30-2 | T. Fujiwara 160409-15 | 0.613 |  | 24* | F1 |
| 30 | 30-3 | T. Fujiwara 160409-16 |  |  | 44* | Recombinant |
| 30 | 30-4 | T. Fujiwara 160409-17 |  |  | 8 | West |
| 30 | 30-5 | T. Fujiwara 160409-19 | 0.930 | C | 18 | West |
| 30 | 30-6 | T. Fujiwara 160409-20 |  |  | 24* | F1 |
| 30 | 30-7 | T. Fujiwara 160409-21 |  | A | 3 | East |
| 31 | 31-1 | T. Fujiwara 170114-51 |  | C | 1 | West |
| 31 | 31-2 | T. Fujiwara 170114-53 |  |  | 28 | East |
| 31 | 31-3 | T. Fujiwara 170114-54 |  | C | 2 | East |
| 32 | 32-1 | T. Fujiwara 170114-1 |  | A | 38 | West |
| 32 | 32-2 | T. Fujiwara 170114-4 |  |  | 41 | Recombinant |
| 32 | 32-3 | T. Fujiwara 170114-5 |  | C | 3 | East |
| 32 | 32-4 | T. Fujiwara 170114-7 |  |  | 3 | East |
| 33 | 33-1 | T. Fujiwara 170114-43 |  |  | 43 | Recombinant |
| 33 | 33-2 | T. Fujiwara 170114-44 |  |  | 1 | West |
| 33 | 33-3 | T. Fujiwara 170114-45 |  | C | 1 | West |
| 33 | 33-4 | T. Fujiwara 170114-46 |  | C | 3 | East |
| 34 | 34-1 | T. Fujiwara 160506-1 |  |  | 1 | West |
| 34 | 34-2 | T. Fujiwara 160506-2 |  |  | 1 | West |
| 34 | 34-3 | T. Fujiwara 160506-3 |  | C | 1 | West |
| 34 | 34-4 | T. Fujiwara 160506-4 |  |  | 7 | West |
| 34 | 34-5 | T. Fujiwara 160506-5 |  |  | 1 | West |
| 34 | 34-6 | T. Fujiwara 160506-6 |  |  | 7 | West |
| 34 | 34-7 | T. Fujiwara 160506-7 |  |  | 1 | West |
| 35 | 35-1 | T. Fujiwara 160506-9 |  |  | 49 | West |
| 35 | 35-2 | T. Fujiwara 160506-10 |  | C | 17 | West |
| 35 | 35-3 | T. Fujiwara 160506-11 |  |  | 7 | West |
| 35 | 35-4 | T. Fujiwara 160506-12 |  |  | 39* | West |
| 36 | 36-1 | T. Fujiwara 160509-1 |  | C | 9 | West |
| 36 | 36-2 | T. Fujiwara 160509-2 |  |  | 9 | West |
| 36 | 36-3 | T. Fujiwara 160509-3 |  |  | 9 | West |
| 36 | 36-4 | T. Fujiwara 160509-4 |  |  | 9 | West |
| 37 | 37-1 | T. Fujiwara 170715-1 |  | C | 1 | West |
| 37 | 37-2 | T. Fujiwara 170715-2 |  |  | 10* | F1 |
| 37 | 37-3 | T. Fujiwara 170715-3 |  |  | 10* | F1 |
| 37 | 37-4 | T. Fujiwara 170715-4 |  |  | 1 | West |
| 37 | 37-5 | T. Fujiwara 170715-7 |  |  | 1 | West |
| 38 | 38-1 | T. Fujiwara 150528-3 |  | C | 1 | West |
| 38 | 38-2 | T. Fujiwara 150528-4 |  |  | 46 | West |
| 38 | 38-3 | T. Fujiwara 150528-5 |  |  | 1 | West |
| 39 | 39-1 | T. Fujiwara 170718-1 |  |  | 36* | F1 |
| 39 | 39-2 | T. Fujiwara 170718-3 | 0.957 |  | 10* | F1 |
| 39 | 39-3 | T. Fujiwara 170718-4 | 0.981 | C | 1 | West |
| 39 | 39-4 | T. Fujiwara 170718-5 |  |  | 1 | West |
| 39 | 39-5 | T. Fujiwara 170718-6 | 0.881 |  | 10* | F1 |
| 39 | 39-6 | T. Fujiwara 170718-7 |  |  | 1 | West |
| 39 | 39-7 | T. Fujiwara 170718-8 |  |  | 1 | West |
| 39 | 39-8 | T. Fujiwara 170718-9 |  |  | 1 | West |
| 39 | 39-9 | T. Fujiwara 170718-10 |  |  | 1 | West |
| 40 | 40-1 | T. Fujiwara 170706-25 | 0.587 |  | 10* | F1 |
| 40 | 40-2 | T. Fujiwara 170706-26 |  | C | 1 | West |
| 40 | 40-3 | T. Fujiwara 170706-27 | 0.381 |  | 10* | F1 |
| 40 | 40-4 | T. Fujiwara 170706-28 | 0.992 |  | 1 | West |
| 40 | 40-5 | T. Fujiwara 170706-30 |  |  | 1 | West |
| 40 | 40-6 | T. Fujiwara 170706-31 |  |  | 1 | West |
| 40 | 40-7 | T. Fujiwara 170706-32 |  |  | 1 | West |
| 40 | 40-8 | T. Fujiwara 170706-33 |  |  | 1 | West |
| 41 | 41-1 | T. Fujiwara 170706-14 |  |  | 1 | West |
| 41 | 41-2 | T. Fujiwara 170706-15 |  | C | 1 | West |
| 41 | 41-3 | T. Fujiwara 170706-16 |  |  | 1 | West |
| 41 | 41-4 | T. Fujiwara 170706-17 |  |  | 10* | F1 |
| 41 | 41-5 | T. Fujiwara 170706-19 |  |  | 1 | West |
| 41 | 41-6 | T. Fujiwara 170706-20 |  |  | 1 | West |
| 41 | 41-7 | T. Fujiwara 170706-21 |  |  | 1 | West |
| 41 | 41-8 | T. Fujiwara 170706-22 |  |  | 7 | West |
| 42 | 42-1 | T. Fujiwara 170706-2 |  |  | 1 | West |
| 42 | 42-2 | T. Fujiwara 170706-3 |  | D1 | 2 | East |
| 42 | 42-3 | T. Fujiwara 170706-4 |  | C | 1 | West |
| 42 | 42-4 | T. Fujiwara 170706-5 |  |  | 1 | West |
| 42 | 42-5 | T. Fujiwara 170706-6 |  |  | 3 | East |
| 42 | 42-6 | T. Fujiwara 170706-7 |  |  | 2 | East |
| 42 | 42-7 | T. Fujiwara 170706-8 |  |  | 1 | West |
| 42 | 42-8 | T. Fujiwara 170706-9 |  |  | 2 | East |
| 42 | 42-9 | T. Fujiwara 170706-10 |  |  | 9 | West |
| 42 | 42-10 | T. Fujiwara 170706-11 |  |  | 2 | East |
| 43 | 43-1 | T. Fujiwara 170705-10 |  | C | 2 | East |
| 43 | 43-2 | T. Fujiwara 170705-11 |  |  | 2 | East |
| 43 | 43-3 | T. Fujiwara 170705-12 |  | C | 1 | West |
| 43 | 43-4 | T. Fujiwara 170705-13 |  |  | 2 | East |
| 43 | 43-5 | T. Fujiwara 170705-14 |  |  | 47 | Recombinant |
| 44 | 44-1 | T. Fujiwara 170703-1 |  |  | 10* | F1 |
| 44 | 44-2 | T. Fujiwara 170703-3 |  |  | 1 | West |
| 44 | 44-3 | T. Fujiwara 170703-4 |  | C | 1 | West |
| 44 | 44-4 | T. Fujiwara 170703-5 |  |  | 42 | Recombinant |
| 44 | 44-5 | T. Fujiwara 170703-6 |  |  | 12 | Recombinant |
| 45 | 45-1 | T. Fujiwara 170704-1 |  | C | 1 | West |
| 45 | 45-2 | T. Fujiwara 170704-2 |  |  | 25* | West |
| 45 | 45-3 | T. Fujiwara 170704-3 |  |  | 1 | West |
| 45 | 45-4 | T. Fujiwara 170704-4 |  |  | 1 | West |
| 45 | 45-5 | T. Fujiwara 170704-5 |  |  | 7 | West |
| 46 | 46-1 | T. Fujiwara 150326-1 |  | C | 1 | West |
| 46 | 46-2 | T. Fujiwara 150326-2 |  |  | 1 | West |
| 46 | 46-3 | T. Fujiwara 150326-3 | 0.978 |  | 1 | West |
| 46 | 46-4 | T. Fujiwara 150326-4 | 1.000 |  | 20* | West |
| 46 | 46-5 | T. Fujiwara 150326-5 |  |  | 1 | West |
| 46 | 46-6 | T. Fujiwara 150326-6 | 1.000 |  | 20* | West |
| 46 | 46-7 | T. Fujiwara 150326-7 |  |  | 1 | West |
| 46 | 46-8 | T. Fujiwara 150326-8 | 0.953 |  | 20* | West |
| 46 | 46-9 | T. Fujiwara 150326-9 |  |  | 51 | West |
| 46 | 46-10 | T. Fujiwara 150326-10 |  |  | 1 | West |
| 47 | 47-1 | T. Fujiwara 150326-14 |  |  | 7 | West |
| 47 | 47-2 | T. Fujiwara 150326-15 |  | C | 8 | West |
| 47 | 47-3 | T. Fujiwara 150326-16 |  |  | 7 | West |
| 47 | 47-4 | T. Fujiwara 150326-17 |  |  | 8 | West |
| 47 | 47-5 | T. Fujiwara 150326-19 |  |  | 8 | West |
| 47 | 47-6 | T. Fujiwara 150326-20 |  |  | 50* | West |
| 48 | 48-1 | T. Fujiwara 150325-2 |  | A | 1 | West |
| 48 | 48-2 | T. Fujiwara 150325-3 |  |  | 1 | West |
| 48 | 48-3 | T. Fujiwara 150325-4 |  |  | 1 | West |
| 48 | 48-4 | T. Fujiwara 150325-5 | 0.984 |  | 1 | West |
| 48 | 48-5 | T. Fujiwara 150325-7 | 0.992 |  | 25* | West |
| 48 | 48-6 | T. Fujiwara 150325-8 |  |  | 1 | West |
| 48 | 48-7 | T. Fujiwara 150325-9 |  |  | 1 | West |
| 48 | 48-8 | T. Fujiwara 150325-10 |  |  | 7 | West |
| 49 | 49-1 | T. Fujiwara 150325-14 |  |  | 1 | West |
| 49 | 49-2 | T. Fujiwara 150325-15 |  |  | 7 | West |
| 49 | 49-3 | T. Fujiwara 150325-17 |  |  | 1 | West |
| 49 | 49-4 | T. Fujiwara 150325-18 |  |  | 1 | West |
| 49 | 49-5 | T. Fujiwara 150325-19 |  |  | 7 | West |
| 49 | 49-6 | T. Fujiwara 150325-20 |  | A | 1 | West |
| 49 | 49-7 | T. Fujiwara 150325-21 |  |  | 1 | West |
| 49 | 49-8 | T. Fujiwara 150325-22 |  |  | 1 | West |
| 49 | 49-9 | T. Fujiwara 150325-23 |  |  | 1 | West |
| 49 | 49-10 | T. Fujiwara 150325-24 |  |  | 1 | West |
| 49 | 49-11 | T. Fujiwara 150325-25 |  |  | 1 | West |
| 50 | 50-1 | T. Fujiwara 150324-1 | 0.943 | B | 7 | West |
| 50 | 50-2 | T. Fujiwara 150324-2 |  |  | 1 | West |
| 50 | 50-3 | T. Fujiwara 150324-5 |  |  | 1 | West |
| 50 | 50-4 | T. Fujiwara 150324-6 |  |  | 1 | West |
| 50 | 50-5 | T. Fujiwara 150324-9 |  |  | 1 | West |
| 51 | 51-1 | T. Fujiwara 160712-1 |  | C | 1 | West |
| 51 | 51-2 | T. Fujiwara 160712-2 |  |  | 1 | West |
| 51 | 51-3 | T. Fujiwara 160712-3 |  |  | 1 | West |
| 51 | 51-4 | T. Fujiwara 160712-4 |  |  | 1 | West |
| 51 | 51-5 | T. Fujiwara 160712-5 |  |  | 1 | West |
| 51 | 51-6 | T. Fujiwara 160712-6 |  |  | 1 | West |
