# Supplementary material for: Independent allopatric polyploidizations shaped the geographical structure and initial stage of reproductive isolation in an allotetraploid fern, Lepisorus nigripes (Polypodiaceae)
Source: PLoS One. 2020 May 20;15(5):e0233095. doi: 10.1371/journal.pone.0233095 (PMC7239481; doi:10.1371/journal.pone.0233095)
Supplement: S5 Table — (DOC) [file pone.0233095.s009.doc]

**S5 Table.** Information for sample ID, voucher specimen, multilocus genotype, plastid haplotype, and spore fertility of *Lepisorus nigripes.* In type column, each sample was classified into East, West, F_1_ and Recombinant based on the result in InStruct (see Materials and Methods and Result in the article).

|  |  | **K =2** | **K =3** | **K =4** | **K =5** | **K =6** | **K =7** | **K =8** | **K =9** | **K =10** |
| --- | --- | --- | --- | --- | --- | --- | --- | --- | --- | --- |
| **Full dataset** | delta K | 0.9621 | 0.0755 | 0.0341 | 0.0825 | 0.0228 | 0.0034 | 0.0012 | 0.0033 | 0.0010 |
|  | MedMeaK | 2 | 3 | 4 | 4 | 3 | 0 | 1 | 0 | 0 |
|  | MaxMeaK | 2 | 3 | 4 | 5 | 3 | 1 | 1 | 1 | 0 |
|  | MedMedK | 2 | 3 | 4 | 5 | 4 | 1 | 1 | 0 | 0 |
|  | MaxMedK | 2 | 3 | 4 | 5 | 4 | 4 | 3 | 1 | 1 |
| **Trimmed dataset** | delta K | 2.1569 | 0.0796 | 0.1978 | 0.0810 | 0.01472 | 0.0225 | 0.0064 | 0.0106 | 0.0038 |
|  | MedMeaK | 2 | 3 | 4 | 2 | 0 | 0 | 0 | 0 | 0 |
|  | MaxMeaK | 2 | 3 | 4 | 2 | 0 | 0 | 0 | 0 | 0 |
|  | MedMedK | 2 | 3 | 4 | 2 | 1 | 0 | 0 | 0 | 0 |
|  | MaxMedK | 2 | 3 | 4 | 2 | 1 | 0 | 0 | 0 | 0 |
